# Meal timing entrains circadian rhythms in the dorsal vagal complex, a brainstem satiety centre

**DOI:** 10.1371/journal.pbio.3003936

**Published:** 2026-08-04

**Authors:** Lukasz Chrobok, Charlotte Muir, Tanya Chonkria Kaur, Emily Birt, Iliana Veneri, Jingyi Kathy Li, Timna Hitrec, Michael Ambler, Anthony Edward Pickering, Hugh David Piggins

**Affiliations:** 1 School of Physiology, Pharmacology, and Neuroscience, Faculty of Life Sciences, University of Bristol, Bristol, United Kingdom; 2 Department of Biomedical and Neuromotor Sciences, University of Bologna, Bologna, Italy; National Institutes of Health, UNITED STATES OF AMERICA

## Abstract

The dorsal vagal complex (DVC) includes a multi-component brainstem satiety centre which has gained attention as a key target of anti-obesity pharmacotherapies. Our recent studies revealed its circadian timekeeping properties, with molecular and electrophysiological 24 h rhythms persisting independently of the primary hypothalamic clock. However, the factors entraining these brainstem oscillators and the downstream transcriptional targets of the DVC molecular clock remain unclear. Here, using PERIOD2::LUCIFERASE reporter mice and fluorescent in situ hybridisation, we quantitatively demonstrate rhythms in core clock gene expression in the caudal DVC *ex vivo* and in vivo. We show that the molecular clock is associated with rhythmic expression of numerous neurotransmitter receptor genes in the DVC in vivo, with the phase of both clock and clock-controlled gene expression tightly regulated by meal timing. These findings uncover food-entrained circadian rhythms in the DVC and have important implications for clinical studies targeting brainstem satiety mechanisms.

## Abstract 2

The dorsal vagal complex (DVC) encompasses a multi-component brainstem satiety center, and exhibits rhythmic activity that is independent of the primary hypothalamic clock. This study examines the factors that entrain these brainstem oscillations, revealing that circadian rhythms in the DVC are entrained by meal timing.

## Introduction

Feeding is a vital behaviour for survival, and organisms have evolved complex mechanisms to ensure adequate food intake. In mammals, neural circuits that regulate feeding are finely tuned to nutrient composition and the daily patterns of food availability in the environment. This allows organisms to not only respond to food presence but also anticipate its availability [1–3].

The suprachiasmatic nucleus (SCN) of the hypothalamus is the central circadian clock in mammals, generating ~24-h rhythms in physiology and behaviour. The SCN’s timekeeping ability is driven by the rhythmic expression of molecular clock components. These core clock genes operate within transcription-translation feedback loops (TTFL) to maintain a cycle close to 24 h. Their protein products are transcription factors that in turn regulate the expression of numerous clock-controlled genes to ensure timely transcription and availability of proteins including signalling molecules, neurotransmitter receptors, and ion channels [4,5].

SCN neurons can adjust the phase of the molecular clock in response to environmental light, via their innervation by the retina, and thus constitute a light-entrainable oscillator [6]. However, under time-restricted feeding (TRF), where food is provided at specific times, feeding becomes an additional, competing time cue (Zeitgeber). Evidence indicates that food anticipatory activity (FAA), the behavioural arousal preceding repetitive timed food presentation, operates largely independent of the SCN and is driven by a network of extra-SCN oscillators in the brain and peripheral organs, collectively termed the food-entrainable oscillator (FEO) [7–10]. Despite extensive research, the FEO has not been localised to a single brain region or peripheral tissue, with several brain centres involved in feeding behaviour identified as contributors [11].

While circadian rhythms of feeding centres and their entrainment to feeding have been extensively studied in the hypothalamus [12–14], we have identified that the dorsal vagal complex (DVC) in the hindbrain exhibits robust circadian timekeeping properties [15]. However, the functional output of the DVC’s molecular clock in regulating transcription of other genes remains unknown. The DVC is a crucial hub for ingestive, metabolic, cardiovascular, respiratory and other homeostatic functions, serving as both a primary source and recipient of vagal innervation [16,17]. Known in the metabolic control field as the brainstem satiety centre, the DVC has recently gained attention for its fundamental role in the action of obesity medications [18–20]. The DVC comprises three anatomically and functionally distinct structures, all expressing circadian rhythmicity: the area postrema (AP; the sensor of blood-borne cues), the nucleus of the solitary tract (NTS; the processor and main receiver of vagal afferents), and the dorsal motor nucleus of the vagus (DMV; the peripheral output). Additionally, we have recently found that nonneuronal ependymal cells lining the central canal and the fourth ventricle within the DVC rhythmically express molecular clock genes *ex vivo* [15]. Although function in the DVC varies along its rostro-caudal axis, our earlier findings point to robust rhythms in a neuroanatomically restricted portion of the DVC that is implicated in the control of food intake [15]. Despite clear evidence implicating the DVC in meal termination and more broadly in feeding behaviour, it is still unclear if and how food intake acts as a Zeitgeber for the DVC circadian oscillators.

Previous studies, including our own, have demonstrated that circadian timekeeping in the DVC of mice and rats is highly sensitive to diet composition [21–23]. The aim of this study was to evaluate whether the circadian properties of the metabolically-relevant caudal DVC at the level of the AP are sensitive to the timing of food intake, to examine the consequences of altered feeding schedules on gene expression within the DVC, and to investigate the contribution of a functional clock gene expression in this brainstem centre to meal architecture and patterning. By investigating conditions in which there are competing Zeitgebers, our experiments show that the DVC’s circadian rhythms are responsive to the timing of food intake, rather than light-dark (LD) cycles. We further show that DVC’s molecular clock influences meal length, especially during the light phase, but is not crucial for the expression of FAA. These food-entrained rhythms in components of the molecular clock further shape the rhythmic transcription of clock-controlled genes, thus aligning DVC gene expression to feeding behaviour.

## Results

### Circadian timekeeping in the caudal DVC

The DVC integrates cardiovascular, ingestive, and gustatory signals, with a functional and anatomical organisation along its rostro-caudal axis. The caudal DVC at the level of the AP processes metabolic and feeding-related cues and forms a brainstem satiety centre [24,25]. In our previous study using coronal brainstem slices from PERIOD2::LUCIFERASE (PER2::LUC) reporter mice, we localised PER2 expression to the AP and subjacent NTS, but only in the caudal portion at the level of the AP (−7.3 to −7.8 mm from bregma [15,26]).

**Fig 1:**
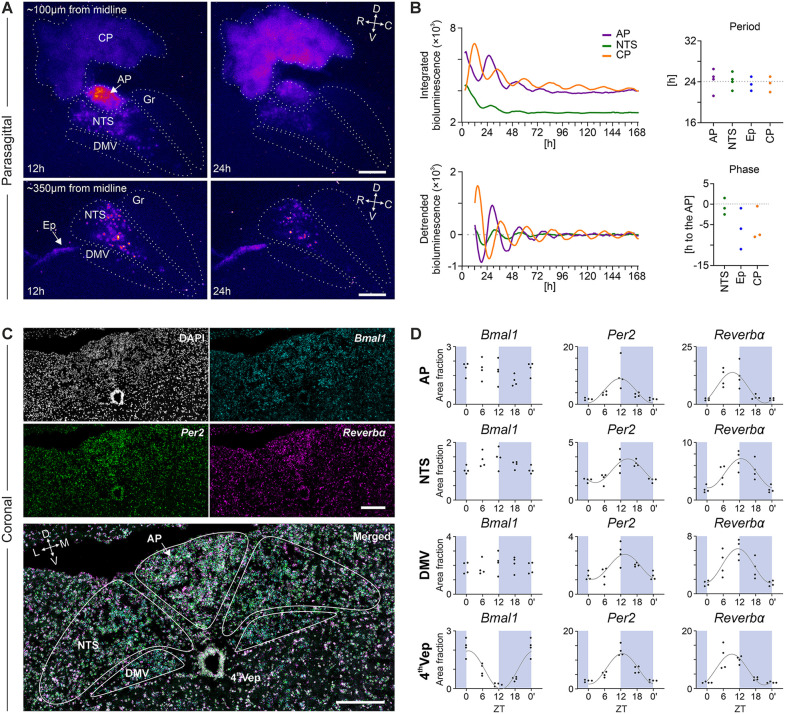
Molecular clock expression in the caudal DVC at the level of the AP under *ad libitum* feeding. **(A)** False-coloured bioluminescence images of parasagittal DVC at two levels from midline, 12 h and 24 h post-start of culture. Borders of three regions of interest (ROIs) were delineated and overlaid. **(B)** Example of raw bioluminescence traces from one parasagittal PER2::LUC brainstem slice (presented in *A – top* panels) over seven days in culture. Twenty-four h-detrended traces are showed below. **(C)** Analysis of period and phase differences relative to the AP in five studied slices. Note, that different DVC substructures were studied in slices obtained from different medio-lateral levels. Each datapoint represent a measurement from one culture. **(D)** Representative confocal microphotograph showing RNAscope images used for these analyses. DAPI – in grey, *Bmal1* – cyan, *Per2* – green, and *Reverbα* (magenta). Merged signal is shown below. **(E)** Sine-wave fitted area fraction measurements of rhythmic core clock gene expression in the AP, NTS, DMV, and 4thVep. All white bars depict 200 µm. The underlying data for this figure can be found in S1 Data.

To confirm and expand upon these findings, here we prepared five parasagittal DVC slices from four PER2::LUC mice and cultured them for seven days *ex vivo* to visualise the rostro-caudal extent of bioluminescence. Three slices were recorded near (within ~100 µm of) the midline, while two were collected 200–350 µm laterally from obex (Fig 1A). Rhythmic PER2 expression was observed throughout the rostro-caudal extent of the AP and in the subjacent NTS (Fig 1A and 1B), but in contrast, the NTS regions rostral to the AP lacked detectable bioluminescence. Sparse PER2-expressing cells were identified in the NTS immediately caudal to the AP. Additionally, parasagittal sectioning allowed us to record bioluminescence from the choroid plexus (CP), a well-recognised extra-SCN oscillator [27,28] attached to the rostral extent of the AP. Nonneuronal PER2::LUC rhythms were also detected in the ependymal layer lining the fourth ventricle and central canal, with circadian periods observed across all DVC oscillators (Fig 1C). Notably, nonneuronal rhythms were phase-delayed relative to those in the AP, but the phase differences in parasagittal plane (Fig 1C) were not as consistent as in coronal slices, as previously reported [15,29,30]. Together, these findings establish the spatial distribution of circadian timekeeping within the caudal DVC, guiding our subsequent in vivo investigations.

Previous qPCR studies [23], including our own [15], have demonstrated clock gene expression in the DVC in vivo. To achieve higher spatial resolution and precise delineation of DVC substructures, we employed RNAscope fluorescent in situ hybridisation technology. Mice (*n* = 16) maintained on *ad libitum* feeding were culled at four Zeitgeber time (ZT) points (ZT0, 6, 12, and 18; *n* = 4 per time point; ZT0 = lights-on). Following our observation of circadian timekeeping in the DVC at the AP level, coronal sections −7.4 mm to −7.6 mm from bregma were used to quantify *Bmal1*, *Per2*, and *Reverbα* expression (Fig 1D) [23]. The superior spatial resolution of RNAscope over qPCR enabled gene expression analysis in the AP, NTS, DMV, and the ependymal lining of the fourth ventricle (Fig 1D). Sine wave fitting confirmed significant daily rhythmicity in *Per2* and *Reverbα* expression across all DVC subregions (*p* < 0.05, CircWave v1.4; Fig 1E), while *Bmal1* rhythms were only significant in the ependymal layer. Interestingly, in contrast to our *ex vivo* recordings, in vivo clock gene rhythms were synchronised in phase across neuronal and nonneuronal DVC oscillators. These results demonstrate that the caudal DVC at the AP level harbours robust molecular circadian timekeeping *ex vivo* which can also be detected in vivo.

### Daily rhythms in the transcriptional programme of the DVC

**Fig 2:**
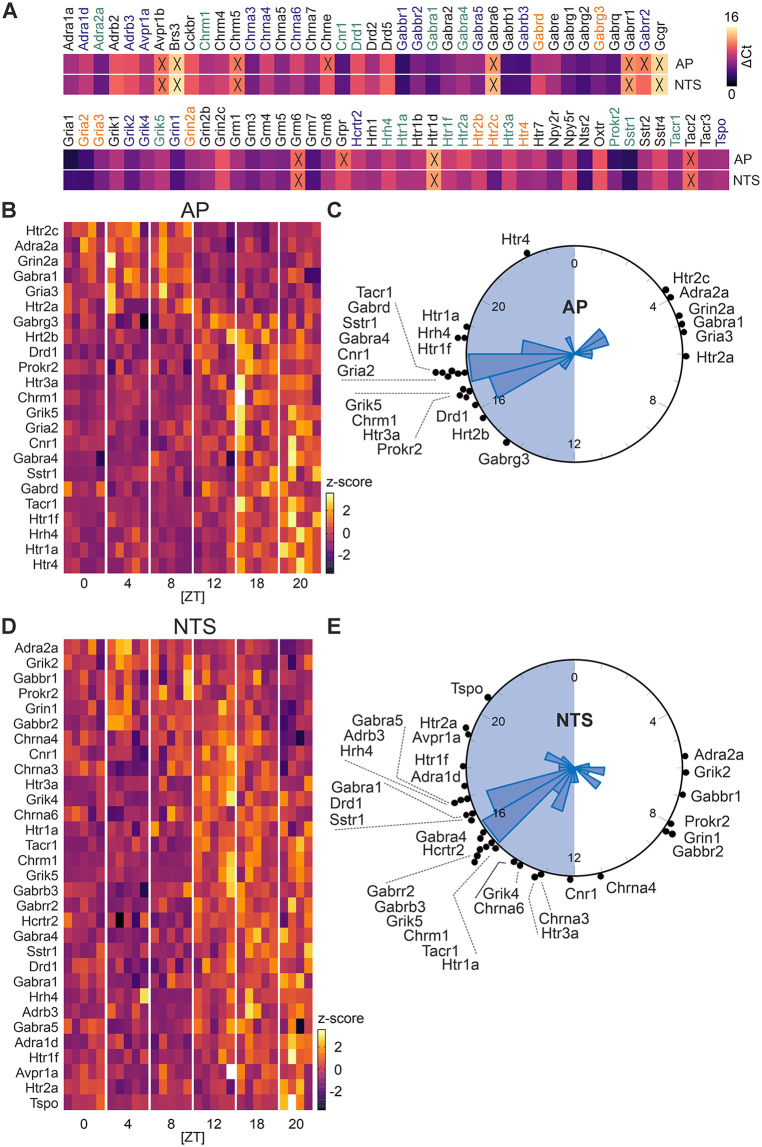
Daily regulation of the transcription of neurotransmitter receptors in the DVC. **(A)** Heatmap showing expression levels of investigated transcripts in the AP and NTS averaged over 24 h. Low ΔCt values (Ct^*gene*^-Ct^*Gapdh*^; dark purple) depict high transcript expression, whereas high ΔCt (light yellow) – low expression. × symbol marks transcripts which did not cross the detection level (Ct^*gene*^>35). Gene names in orange followed significant rhythmic expression over 24 h in the AP, in purple – in the NTS, and those in green were common for both structures. **(B, D)** Heatmaps depicting z-scored expression levels of all rhythmic gene (each box within the row represents one mouse), ordered top-to-bottom by their acrophase. **(C, E)** Raleigh plots displaying acrophases for rhythmic genes in the AP and in the NTS, with circular histograms superimposed (bin = 1 h, bar size represents number of genes peaking at that hour). AP – area postrema, NTS – nucleus of the solitary tract. Raw data are presented in S1 Fig. The underlying data for this figure can be found in S1 Data.

Clock gene proteins serve as transcriptional factors to orchestrate the timely expression of downstream genes over ~24 h. Notably, in the SCN and other extra-SCN oscillators, the transcription of genes for receptors of neurotransmitters and neuromodulators display robust daily and circadian rhythmicity [31–33]. Therefore, we directed our investigation towards delineating the expression of neurotransmitter receptors in tissue punches obtained from the AP and NTS at 4 h intervals over a 24 h period (*n* = 5/time point). We assessed the expression levels of 84 RNA transcripts in each sample, of which 72 and 75 were found to be reliably expressed (Ct < 35) in the AP and NTS, respectively (Fig 2A). Subsequently, by fitting sine waves to expression profiles normalised to *Gapdh* (housekeeping gene without clear circadian variation) across six daily timepoints, we identified 23 transcripts as rhythmically expressed over 24 h in the AP and 32 in the NTS. A total of 15 transcripts exhibited daily rhythmicity common to both regions (Figs 2A and S1). Intriguingly, extrapolated peak times for these rhythmic transcripts in the AP were predominantly clustered during the middle of the night (ZT16–18), with a secondary cluster observed in the early part of the day (ZT4–6; Fig 2B and 2C). A similar clustering was observed for rhythmic transcripts in the NTS (Fig 2D and 2E).

Rhythmic transcripts in the AP and NTS were further explored for the presence of putative E-boxes [34], and thus their potential to be directly responsive to the transcriptional activity of the molecular clock. Motif analysis assessing BMAL1 binding profile (taken from JASPAR database) revealed that 30 out of 34 rhythmic transcripts analysed contained putative E-boxes (S2 Fig). The remaining 4 genes with rhythmic transcripts (*Grik5*, *Hrt2b*, *Prok2*, and *Ssrt1*) detected with our RT^2^ Profiler PCR Arrays must be either controlled by transcription factors other than BMAL1/CLOCK or their daily oscillations are a result of rhythmic input to the DVC. Four rhythmic genes (*Adra1d*, *Adrb3*, *Grin2a*, and *Hrh4*) were excluded from the analysis, as their promoter sequences were not known or listed in the eukaryotic promoter database [35–37].

Previously, we developed a model (based on recordings of rhythmic PER2::LUC expression in DVC tissue explants) which predicted that the circadian oscillators in the DVC would be malleable and readily reset by exogenous cues [29]. We therefore investigated whether increased expression of neurotransmitter receptor genes in the AP and NTS is a direct consequence of feeding during the active phase of mice, commencing at the onset of the night. For this aim, we compared two cohorts of 5 animals. One group was culled immediately after undergoing a single 6 h-long restriction of food availability from the late day (ZT10) to early night (ZT16). The second group experienced the same 6 h food restriction followed by 2 h of unlimited access to chow and thus was culled in the middle of the night (ZT18). Interestingly, this transient fasting/refeeding regimen had minimal impact on overall neurotransmitter gene expression levels in the DVC. In the AP, only 9 out of 72 transcripts tested were affected by food restriction and/or refeeding, of which just 3 exhibited daily rhythmicity in the previous experiment (S3 Fig). Similarly, in the NTS, acute changes in food availability affected 11 out of 75 transcripts, with only 5 displaying rhythmic patterns under *ad libitum* conditions (S3 Fig). These findings collectively suggest that the expression of clock genes in the AP and NTS, rather than acute change in feeding behaviour, orchestrate the majority of the phasic regulation of downstream gene expression, including those encoding neurotransmitter and neuropeptide receptors.

### Core clock gene expression in the DVC during TRF

**Fig 3:**
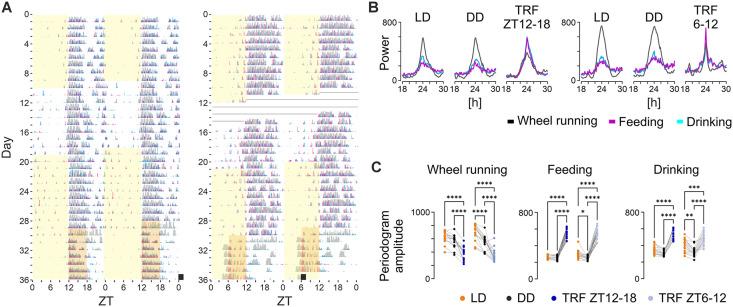
Effects of time-restricted feeding on behavioural rhythms. **(A)** Representative actograms showing wheel running (in grey), water (blue), and food intake (magenta). Yellow boxes depict light phase; note the persistence of behavioural rhythms in constant darkness. Straight line codes for missing data. Food was presented *ad libitum* until day 29, where 6 h-long time-restricted feeding (TRF) protocol started; food presentation time is then depicted by an orange box. **(B)** Representative periodograms showing the robustness of ~24 h rhythms in all three behaviours under light-dark (LD), constant darkness (DD), and TRF conditions. **(C)** Repeated-measures comparisons showing that TRF significantly decreases robustness (periodogram amplitude) of the wheel running rhythms, while boosting feeding and drinking rhythms. *p < 0.05, **p < 0.01, ***p < 0.001, ****p < 0.0001, Šídák’s multiple comparisons test. The underlying data for this figure can be found in S1 Data.

In contrast to acute fasting-refeeding protocols, consistent presentation of daily food within a predictable time window over several days induces FAA, indicative of activation of the SCN-independent food-entrainable circadian oscillator (FEO) [9]. To assess how feeding schedules influence DVC timekeeping, we monitored the feeding, drinking, and wheel running behaviours of mice subjected to TRF. Initially, all mice underwent monitoring with *ad libitum* access to food under a 12:12-h LD cycle as well as constant darkness (DD) to establish their daily and circadian behavioural profiles. Subsequently, following re-entrainment to LD, mice were subjected to TRF for 6–7 days, either during the initial 6 h of the dark phase (ZT12–18, *n* = 16) or the late portion of the light phase (ZT6–12, *n* = 16; Fig 3A). In both TRF groups, significant enhancement in the rhythmicity of feeding and drinking patterns was observed (Fig 3B and 3C), while wheel running rhythms exhibited reduced robustness due to fragmentation of daily locomotor activity between FAA and nocturnal wheel running (Fig 3B and 3C).

FAA was significantly reduced in night-fed compared to day-fed animals (*p* = 0.0134, Sidak multiple comparisons test, S4 Fig). Indeed, only 4 of 16 night-fed mice displayed notable FAA, consistent with feeding at the physiological time of day attenuating the anticipatory drive [38]. Nocturnal wheel running was unaffected by feeding schedule (*p* = 0.1847, Sidak multiple comparisons test, S4 Fig), highlighting the behavioural independence of SCN-driven nocturnal activity and FEO-driven FAA. As predicted, food intake was significantly higher in night-fed animals (*p* = 0.0074, *t* test, S4 Fig). Despite this, no significant differences in body weight change following TRF were observed between the late-day and early-night fed cohorts (*p* = 0.4778, *t* test, S4 Fig).

**Fig 4:**
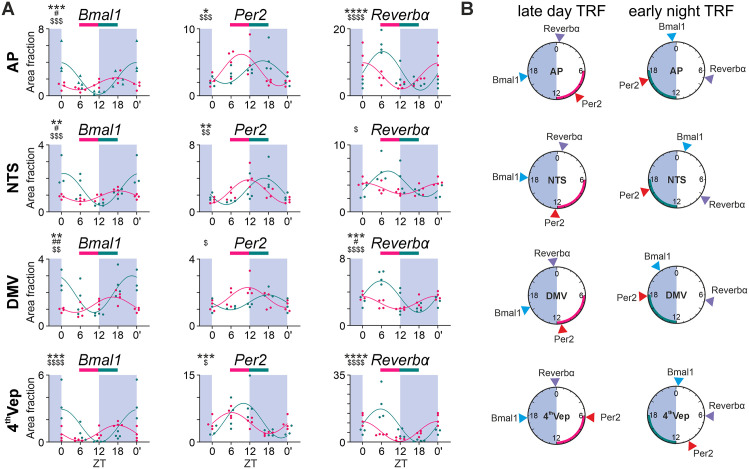
The phase of core clock genes expression in the DVC in vivo follows the time of food presentation. **(A)** Sine-wave fitted area fraction measurements of rhythmic core clock gene expression in the AP, NTS, DMV, and 4thVep. Magenta – late day ZT6-12 time-restricted feeding (TRF), green – early night ZT12-18 TRF. 2-way ANOVA results are depicted by * - time of day, # - feeding time, $ - interaction, where *p < 0.05, **p < 0.01, ***p < 0.001. **(B)** Raleigh plots displaying acrophases for core clock genes in these two TRF conditions, where the arches depict the time of food presentation. Note that peak expression of these core clock genes is better predicted by feeding time than light-dark cycle. AP – area postrema, NTS – nucleus of the solitary tract, DMV – dorsal motor nucleus of the vagus, 4thVep – ependymal cell layer lining the 4th ventricle/central canal. The underlying data for this figure can be found in S1 Data.

To elucidate the pattern of core clock gene expression in the DVC under these competing food and light Zeitgeber conditions, we culled mice following the same regimes of TRF at four daily timepoints (ZT0, 6, 12, and 18; *n* = 4 each) and conducted RNAscope in situ hybridisation on DVC brain slices to delineate the spatiotemporal expression patterns of three core clock genes: *Bmal1*, *Per2*, and *Reverbα*. Irrespective of the timing of food presentation in TRF, all core clock genes exhibited rhythmic expression across all four regions of the DVC: the AP, NTS, DMV, and ependymal lining of the fourth ventricle (Fig 4A). However, their phases of expression aligned with meal timing rather than LD cycles. Notably, across all oscillators, *Reverbα* peaked ~6 h prior to food presentation, while *Bmal1* (arrhythmic under *ad libitum* feeding) peaked ~6 h after food withdrawal (Fig 4B), irrespective of the time set by the LD cycle. Interestingly, *Per2* exhibited a consistent phase across the AP, NTS, and DMV, peaking at the end of the feeding period, whereas in ependymal cells, *Per2* peaked prior to food intake in both TRF conditions (Fig 4A and 4B).

A phase divergence between predominantly neuronal parts of the DVC and ependymal cells has been previously noted under *ex vivo* culture conditions [15,29]. Moreover, when clock gene transcription was examined under *ad libitum* feeding conditions (*n* = 16) over the LD cycle, the phase of *Per2* across DVC regions was synchronised, peaking around the transition from light to dark (S5 Fig). This suggests that meal timing differentially entrains the molecular clock in ependymal cells and the remaining parts of the DVC.

Next, a separate group of animals underwent early-day TRF (ZT0−6, *n* = 16) for 6 days, followed by the same clock gene expression assessment with RNAscope. Surprisingly, this feeding regimen resulted in misalignment of DVC oscillators, leading to cessation of *Bmal1* rhythms in the NTS, and *Per2* rhythms in the AP and DMV (sine wave fit *p* > 0.05; CircWave). With the majority of clock gene expression out of phase with food presentation compared to other TRF timings, only the remaining rhythms in the NTS reliably followed the new mealtime; *Per2* consistently peaked at the end of feeding, and *Reverbα* ~5 h before food presentation (S6 Fig). Similarly, another cohort of 14 mice underwent a TRF at late night (ZT18-0) for 1 week. RNAscope assessment of clock gene expression showed similar, partial misalignment of oscillators with *Bmal1* and *Per2* not significantly rhythmic in the AP, and *Per2* not cycling in the NTS. In this cohort, only *Bmal1* in the NTS and DMV consistently followed the mealtime (S6 Fig).

With the above observation on the phase entrainment of clock gene expression by TRF, we determined a ‘food entrainment error’ (FEE, see *Methods* for description) for each gene and each DVC oscillator in order to measure the degree of entrainment to different TRF schedules (S5 Fig). The values of FEE under late day TRF (ZT6-12) were in the range of ±2 h in all DVC substructures for all three core clock genes tested. This means that their phase can follow a 6 h advance in feeding with relatively high precision. However, under an early day TRF (ZT0-6) and late night TRF (ZT18-0), only the acrophase of most clock gene expression in the NTS (with the exception of *Reverbα* in the late night TRF group) and *Bmal1* in the DMV following the late night feeding showed a FEE in this range, with all other structures exhibiting error in the −2 h to −8 h range (S6 Fig).

Taken together, these data indicate that the phase of clock gene expression in the DVC follows meal presentation time, rather than LD cycles. While the phase of all DVC substructures can be reliably delayed by late-day TRF, only the NTS shows enough flexibility to be advanced in phase after a 6-day long early-day TRF.

### TRF shifts rhythms in the transcriptional programme in the DVC

Clock genes act as transcription factors, regulating the expression of numerous downstream genes to maintain proper 24-h cycles [5]. Our dataset presented above showed that the timing of food presentation serves as a potent entraining factor for DVC molecular clock rhythms. Thus, we investigated whether TRF influences the phase of downstream rhythms in neurotransmitter receptor gene expression.

We subjected another two cohorts of mice to TRF for 1 week, either at the start of the light phase (TRF ZT0-6, *n* = 16, outside of normal feeding time for rodents) or the dark phase (TRF ZT12-18, *n* = 16, within their normal nocturnal feeding window). A range of seven neurotransmitter receptor genes (*Htr2c, Grik5, Gabra4, Gabra1, Adra2a, Prokr2,* and *Sstr1*), previously shown to exhibit robust rhythmicity under *ad libitum* feeding, were selected for analysis. Additionally, we evaluated *Per2* and *Bmal1* expression to examine the phase of the molecular clock under TRF conditions in this cohort of animals. Gene expression was analysed using NanoString nCounter technology.

**Fig 5:**
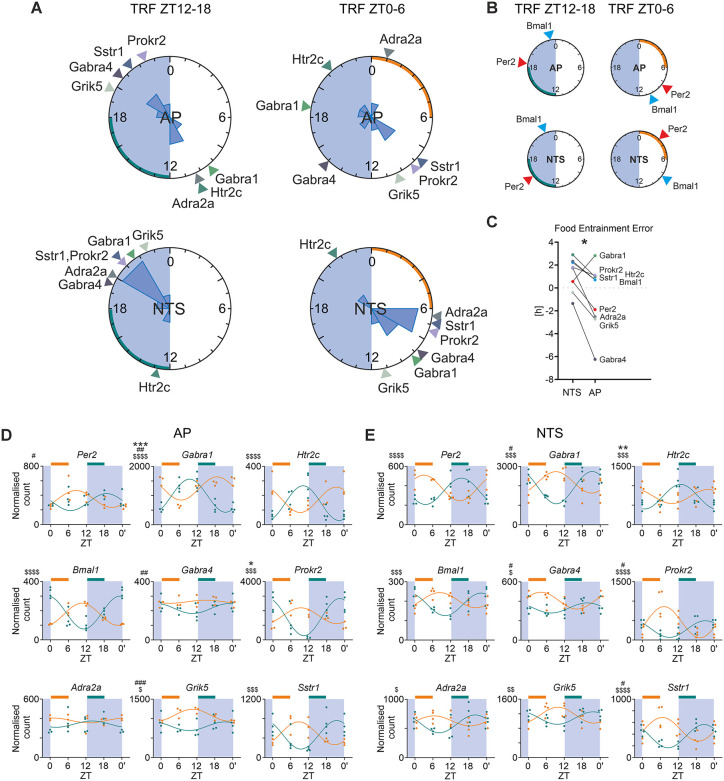
Expression of neurotransmitter receptor genes in the DVC is entrained by feeding time. **(A)** Raleigh plots displaying acrophases for rhythmic neurotransmitter receptor genes in the AP and in the NTS, with circular histograms (bin = 2 h) overlayed. Green and orange arches depict time when food was presented. **(B)** Corresponding Raleigh plots for core clock genes *Bmal1* and *Per2*. **(C)** Food entrainment error for the AP and the NTS in late day TRF condition relative to early night TRF (FEE, observed phase – projected phase); *p < 0.05, paired t *t*est. **(D, E)** Sine-wave fitted normalised counts of rhythmic core clock transcripts in the AP and in the NTS, respectively; a result of NanoString nCounter analysis. 2-way ANOVA results are depicted by * - time of day, # - feeding time, $ - interaction, where *p < 0.05, **p < 0.01, ***p < 0.001 (and accordingly for other depicters). AP – area postrema, NTS – nucleus of the solitary tract, TRF – time-restricted feeding. The underlying data for this figure can be found in S1 Data.

Under these TRF conditions, all selected transcripts displayed significant daily rhythmicity, as determined by sine wave fitting (*p* < 0.05, CircWave; Fig 5A and 5B). In the early-night TRF group, most genes peaked late at night, with the exception of *Gabra1* and *Adra2a* in the AP and *Htr2c* in both the AP and NTS reaching their acrophases in the late day/early night (Fig 5C). This bimodal acrophase distribution mirrored the pattern observed under *ad libitum* conditions (Fig 2B and 2C). Notably, the phase of rhythmic neurotransmitter receptor gene expression shifted according to TRF timing (Fig 5A, 5D and 5E). Under early-day TRF, acrophases aligned more closely with feeding times, with greater precision and clustering in the NTS compared to the AP. This was reflected in a significantly higher FEE for the AP relative to the NTS (*p* = 0.0223, paired t *t*est; Fig 5C). As with the receptor genes, *Per2* and *Bmal1* expression also aligned with feeding schedules (Fig 5B).

Altogether, these findings demonstrate that food-entrained rhythms in molecular clock genes are accompanied by corresponding shifts in the daily oscillations of secondary genes, ensuring that neurotransmitter receptor expression in the DVC is synchronised with feeding behaviour.

### Molecular clock in DVC neurons shapes meal duration but is not essential for FAA

**Fig 6:**
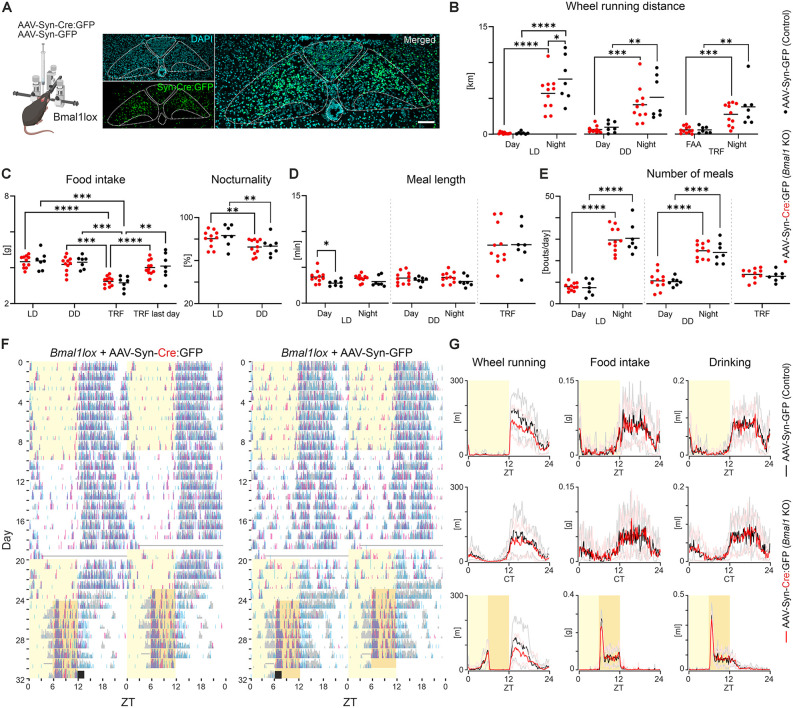
Behavioural phenotyping of the induced *Bmal1*^*KO*^ in DVC neurons. **(A)** Cartoon showing AAV-Syn-Cre:GFP or AAV-Syn-GFP injected into the AP and NTS of Bmal1lox mice. Representative image showing transfection site. Green – Cre:GFP, cyan – DAPI. White bar denotes 100 µm. **(B)** Comparison of wheel running distance per 24 h between the DVC *Bmal1*^*KO*^ mice (red) and controls (black), in 12:12 light-dark cycle (LD), constant darkness (DD) and during the time-restricted feeding protocol (ZT6-12) in LD. FAA – food anticipatory activity. **(C)** Summary of the total food intake per 24 h in LD, DD, TRF, and the last day of TRF, followed by the % of food consumed at night (nocturnality index). **(D)** An average length of feeding bouts. **(E)** Mean number of feeding bouts per 24 h. *p < 0.05, **p < 0.01, ***p < 0.001, ****p < 0.0001, Fisher’s LSD test. **(F)** Representative actograms with wheel running (in grey), water (blue), and food intake (magenta). Yellow boxes depict light phase and orange boxes depict food access during TRF (food was presented *ad libitum* until day 24). Straight line codes for missing data. Note the persistence of FAA under TRF in both *Bmal1*^*KO*^ (*left*) and control mouse (*right*). **(G)** Average plots for each behaviour and condition (LD – *top*, DD – *middle*, and TRF – *bottom*). The underlying data for this figure can be found in S1 Data. Created in BioRender. Chrobok, L. (2026) https://BioRender.com/jdiud7a.

Finally, to determine the contribution of the molecular clock in the DVC to meal architecture (meal length and frequency) and the pattern of food intake across 24 h, we performed stereotaxic injections of either AAV-Syn-Cre:GFP (*n* = 11) or AAV-Syn-GFP (*n* = 7) into the AP and bilaterally into the NTS of 18 Bmal1lox mice (Fig 6A). Expression of Cre resulted in neuron-specific *Bmal1* knock-down and, consequently, a collapse of the molecular clock. After 2 weeks of post-surgical recovery, feeding, drinking, and wheel running behaviours were monitored as described above.

Under *ad libitum* feeding in 12:12 LD conditions, DVC-specific *Bmal1*^*KO*^ mice exhibited reduced nocturnal wheel running activity (*p* = 0.0389, Fisher’s LSD; Fig 6B). This difference, however, was not present under constant darkness (*p* = 0.2732), suggesting it does not arise from an endogenous circadian deficit. Daily food intake (*p* = 0.8059, 2-way ANOVA; Fig 6C) and the proportion of food consumed during the active phase (*p* = 0.5886, 2-way ANOVA; Fig 6C) were comparable between neuronal *Bmal1*^*KO*^ mice and controls in both LD and DD conditions.

Analysis of feeding bouts revealed that average meal duration was increased in *Bmal1*^*KO*^ mice (*p* = 0.0234, 2-way ANOVA), reaching significance during the light phase (*p* = 0.01, Fisher’s LSD; Fig 6D). This finding aligns with the established role of the DVC in regulating meal size and meal termination and suggests that an intact molecular clock within DVC neurons contributes to this control. Meal frequency did not differ between genotypes (*p* = 0.9106, 2-way ANOVA; Fig 6E).

We next subjected the animals to a week-long TRF protocol, with food available only during the late day (ZT6-12; Fig 6F). Strikingly, FAA was unaffected by DVC-specific *Bmal1* deletion (Fig 6F and 6G). No genotype differences were observed in wheel running distance either during FAA or during the subsequent nocturnal activity period (Fig 6B). Total food intake during TRF, as well as meal duration and meal frequency, were also unchanged by the loss of the DVC molecular clock (Fig 6C, 6D and 6E). The reconstruction of AAV injection sides is presented in S7 Fig.

Taken together, these findings indicate that *Bmal1*-dependent clock gene expression in DVC neurons is not required for the generation of FAA but does modulate diurnal mechanisms governing meal termination.

## Discussion

Our study reveals that the DVC demonstrates robust daily properties in vivo, which are highly sensitive to meal timing. This supports the role of feeding schedules as potent Zeitgebers for extra-SCN oscillators including the DVC [8,39]. Under competing photic and nonphotic Zeitgeber conditions, we show that clock gene expression in the DVC is entrained predominantly by TRF, overriding LD cycles. The key finding of this work is that this food entrainment affects the timely transcription of neurotransmitter receptor genes, aligning the DVC transcriptome with feeding behaviour.

The caudal DVC is a critical hub for metabolic, cardiovascular, and ingestive processes essential for survival. At the level of the AP, a component of the NTS contributes specifically to meal termination [16,17,40], whereas the AP contributes to nausea and sickness behaviour [41,42]. Interestingly, the caudal DVC serves as both a source and a primary target of vagal circuitry connecting the gastrointestinal tract and the brain. Disrupting liver-brain synchronisation via the vagal afferents impacts body weight and food intake [43,44]. Intrinsic circadian properties of vagal afferent neurons are also well documented [45–47]. Additionally, neurons within the caudal DVC respond to peri-prandial hormonal cues, further underscoring its role in the gut-brain axis [16,17,40,48]. Our findings reveal that the molecular clock is expressed in all neuronal and nonneuronal substructures of the DVC: the AP, NTS, DMV, and ependymal cell layer of the fourth ventricle and central canal, but only at the specific rostro-caudal division of the DVC at the level of the AP. These findings extend our previous observations [15], demonstrating widespread circadian timekeeping across the DVC whose organisation parallels that of the SCN, where diverse neuronal populations synchronise molecular rhythms to regulate physiological outputs [49]

Our prior *ex vivo* and in vivo investigations, as well as our computational modelling [15,21,22,29,50,51], have established the intrinsic circadian properties of the DVC. However, the environmental cues that entrain the DVC remained unclear until now. In the SCN, synchronised rhythms in molecular clock expression, which maintain daily outputs such as neurophysiological activity and neurohormone release, are entrained predominantly by light [6,52]. In contrast, the DVC lacks direct retinal innervation [53,54] and it has a well-established role in controlling ingestive behaviour [16,17].

Accumulating research has shown that long-term high-fat diet (HFD) disrupts circadian oscillators [55,56], including molecular clock rhythms in the DVC [23]. Moreover, it is well-documented that feeding patterns are altered by consumption of HFD via dysfunction of the molecular clock as well as an increased hedonic drive to eat highly palatable food throughout the 24 h cycle [57]. Our own studies corroborate these findings, showing that even short-term HFD alters rhythmic properties in the DVC *ex vivo* while disrupting feeding patterns, leading to food intake during the behaviourally quiescent light phase [21,22]. Here we show that meal timing is crucial for the phase of clock gene expression in the DVC, which suggests that changes in daily patterns of feeding evoked by HFD, rather than diet composition, contribute to the detrimental effects of HFD on the DVC circadian timekeeping. Therefore, the potential protective effects of TRF during the behaviourally active phase on the rhythmicity of the DVC under HFD conditions warrant further investigation.

Our findings further suggest the inclusion of the DVC within the FEO network. The alignment of DVC clock gene expression to TRF is consistent with patterns observed in hypothalamic feeding centres including the arcuate nucleus and dorsomedial hypothalamus [14,58,59]. Similarly, we found that the TRF is a robust synchroniser of the DVC, markedly enhancing *Bmal1* rhythmicity in the AP, NTS, and DMV, with otherwise weak or undetectable daily rhythms under *ad libitum* feeding conditions. While the SCN-independent nature of FAA is well-established [9], the specific contribution of the DVC has received relatively little attention. Previous work demonstrating that AP lesions fail to abolish FAA [60] reinforces the concept that no single brain region constitutes the entire FEO [11]. Our data strengthen this view: *Bmal1* deletion restricted to AP and NTS neurons did not impair FAA, suggesting that anticipatory behaviour relies on distributed networks and may additionally involve nonneuronal cell populations within the DVC, which warrant further investigation.

The same genetic disruption of the DVC molecular clock moderately altered meal architecture, specifically lengthening meal bouts, but only during the light phase in LD conditions. This phenotype aligns with the established role of the DVC—and particularly the NTS—in sensing and integrating vagal and humoral satiety signals that control meal termination [13]. Loss of *Bmal1* may blunt the normal time-of-day modulation of DVC neuronal excitability and responsiveness to satiety cues, which themselves show circadian variation in efficacy [12]. Consistent with this, our data reveal a daily transcriptional programme in the DVC affecting multiple neurotransmitter receptors that mediate vagal and humoral satiety signalling. Disrupting the molecular clock is therefore likely to flatten these rhythms, reducing the temporal gating of satiety inputs and delaying meal termination, leading to longer meals. Due to a small effect size reported in our study, these experiments should be replicated in longer protocols and during a feeding challenge, e.g., with HFD. Taken together, our findings position the DVC as a flexible and responsive node within the FEO network—one capable of adjusting its transcriptional programme to predict and respond to feeding schedules—while simultaneously contributing to the temporal structure of individual meals.

Interestingly, the timing of TRF within the 24-h cycle affects the degree of DVC entrainment. A late-day TRF schedule (phase advance relative to the active phase) produced more reliable shifts in DVC rhythms compared to early-day or late-night TRF (phase delay). This aligns with behavioural evidence suggesting that circadian rhythms are more readily advanced by nonphotic cues and delayed by photic stimuli [61]. Although a week of TRF is enough to synchronise peripheral clocks to a reversed feeding time [62], longer TRF protocols may enhance entrainment to early-day feeding schedules. Among DVC components, the NTS demonstrated the most precise alignment of clock gene expression under both TRF conditions, suggesting a role for vagal afferent signalling in entraining the DVC circadian timekeeping. Given that gut-derived vagal innervation preferentially targets the NTS [17,63,64], and exhibits circadian rhythmicity itself [45,65], it is plausible that the vagus nerve functions as a key pathway for feeding-driven entrainment, analogous to the role of retinal input in the SCN. Future studies are needed to investigate this hypothesis.

Crucially, these food-entrained molecular clock rhythms orchestrate the rhythmic transcriptional repertoire of the DVC. Under *ad libitum* feeding, we observed daily oscillations in the expression of multiple neurotransmitter receptor genes. Similar temporal clustering or synchronisation of gene expression is reported in other clock gene-expressing peripheral tissues, such as the heart and liver, where the circadian clock governs tissue-specific functions [66]. Our findings also mirror those in the SCN, where the molecular clock’s role in regulating downstream gene expression is well established [4,5]. Importantly, the phase of these gene expression rhythms in the DVC shifted in accordance with clock gene entrainment to altered meal timing. This includes transcripts encoding neurotransmitter receptors with documented roles in the control of feeding (such as *Sstr1, Hrt2c,* and *Cnr1*) [67–69] and gastric mobility (*Gabra1* or *Gabra4*) [70,71]. This highlights the functional output of the DVC’s circadian machinery, shaping the daily transcriptional landscape of its constituent cells to align with environmental feeding cues.

Our findings have important implications for understanding the circadian plasticity of the DVC and its role in metabolic health. Feeding schedules that entrain DVC molecular clocks could optimise the timing of neurotransmitter receptor expression, potentially enhancing the efficacy of pharmacological interventions targeting DVC circuits. Pharmacological agents that target neurotransmitter receptors in the DVC including CNR1, HTR2c, and GLP1R are already in clinical use or under investigation for obesity and diabetes treatment [18,20,72]. Incorporating meal timing into chronopharmacological strategies could synergise with these treatments, utilising the DVC’s circadian rhythmicity to improve therapeutic outcomes.

In conclusion, our study establishes the DVC as an integral node in the FEO network, capable of integrating feeding cues to modulate circadian gene expression. By demonstrating the entrainment of DVC molecular clocks by meal timing, we provide new insights into the neural mechanisms underlying food entrainment and lay the groundwork for circadian-based interventions in metabolic disorders.

## Methods

### Animals

All experiments described in this article were conducted using 172 adult (>8 weeks old) mice of both sexes, on a C57BL/6J genetic background locally bred at the Animal Services Unit at the University of Bristol. Mice were housed at 20–22 °C with around 40% humidity, provided with *ad libitum* access to food (unless otherwise specified) and water before and throughout the experiments, and maintained on a 12:12-h LD cycle in breeding rooms. Animals were also kept under 12:12-h LD conditions during experiments unless otherwise stated. All experimental procedures were approved by the University of Bristol Animal Welfare and Ethics Review Body (AWERB), conducted in accordance with the UK Animals (Scientific Procedures) Act of 1986 and project-specific UK Home Office licence (PP8928230). All measures were taken to refine procedures and minimise animal suffering.

### PERIOD2::LUCIFERASE bioluminescence

#### Tissue preparation.

Four adult male *mPer2Luc* knock-in (PER2::LUC) mice were overdosed with pentobarbital sodium (200 mg/ml, i.p.), and trancardially perfused with ice-hold Hank’s Balanced Salt Solution (HBSS; Sigma, Germany) supplemented with 1 mg/ml penicillin-streptomycin (Gibco Invitrogen Ltd, UK) and 0.01 M HEPES (Sigma). Following decapitation, their brains were promptly removed from the skull into the same ice-cold media and cut into 250 µm parasagittal slices with a vibratome (Camden Instruments, UK). The DVC was identified under a stereomicroscope and dissected with a scalpel. The explants were then transferred to 30 mm Millicell cell culture inserts (Merck, Germany) in glass-coverslip sealed Fluorodish culture dishes (World Precision Instruments, USA) with sterile culture medium (Dulbecco’s Modified Eagle’s Medium; DMEM, Sigma) supplemented with 0.1 mM luciferin (Promega, USA), B27 Plus (Gibco Invitrogen Ltd, USA), 1 mg/ml penicillin-streptomycin (Gibco Invitrogen Ltd), 10 mM HEPES (Sigma), and 3.5 g/L d-glucose (Sigma).

#### Data acquisition and analysis.

Slices were cultured for 7 days and images were taken using the Olympus Luminoview LV200 (Olympus, Japan) System with a cooled Hamamatsu ImageEM C900-13 EM-CCD camera fitted with a 20 × 0.4 NA Plan Apo objective (Olympus, Japan). Throughout the recording, slices cultures were maintained at 37 °C. Exposure time was 30 min and gain was kept constant.

Images were analysed in ImageJ, using a region of interest tool to select whole brain areas for assessing relative bioluminescence over time. The first 6 h of all recordings were excluded and raw data were subject to a 3 h running average smooth before further analysis. Peaks of individual bioluminescence traces of whole structures were determined manually. An average of three peak to peak measurements was used to measure period and phase was determined as a difference in peak time at day 2. Detrending was done by extracting a 24 h running average from each corresponding data point. Statistical analysis was performed in GraphPad Prism 10 (GraphPad Software LCC, MA, USA).

### Fluorescent in situ hybridisation (RNAscope)

#### Tissue preparation.

RNAscope in situ hybridisation was performed to elucidate rhythms in clock gene expression in the DVC of mice undergoing *ad libitum* feeding or 6 h-long TRF for 7 consecutive days. Seventy-eight mice were divided into five feeding groups (*ad libitum*, TRF ZT0-6, TRF ZT6-12, TRF ZT12-18, TRF ZT18-0) and deeply anaesthetised with an overdose of pentobarbital sodium (200 mg/ml, i.p.) and subsequently decapitated in 6-h intervals over 24 h (ZT0, 6, 12, and 18; *n* = 3–4 animals per time point per diet). Brains were promptly removed from the skulls, flash-frozen in Cryomatrix (Epredia, UK) over dry ice, and stored at −80 °C.

All brains were sectioned into 16 μm thick coronal slices at −20 °C using a cryostat (Leica CM1860 UV). Slices were thaw-mounted on Superfrost-Plus slides (Thermofisher, USA) and stored at −80 °C until the day of the protocol. Then, slices were thawed, fixed in 4% paraformaldehyde (PFA) solution in 0.1 M phosphate buffered saline (PBS) for 15 min at room temperature (RT), rinsed twice in fresh PBS, and dehydrated in increasing ethanol concentrations (50%, 70%, 100%, and 100%). Slices were stored at −20 °C in the second 100% ethanol solution overnight.

#### RNAscope protocol and imaging.

First, slides were air dried, and each slice was outlined with a hydrophobic barrier pen. Then, slices were processed according to RNAscope multiplex v2 in situ hybridisation protocol (Advanced Cell Diagnostics—ACD, USA). In brief, slices were incubated at RT with hydrogen peroxide for 10 min and with protease IV for a further 12 min. Slices were then incubated at 40 °C for 2 h with a set of probes: *Arntl, Per2*, and *Nr1d1*. Next, the signal was amplified in a three-step protocol. Finally, three fluorophores (Opal dyes: 520, 570, and 690; Akoya Biosciences, MA, USA) were tagged via a horseradish peroxidase reaction. Slides were air dried, and cover slipped with Fluoroshield containing DAPI (Sigma, Germany).

#### Imaging, analysis, and statistics.

The area containing the DVC was scanned at 40× magnification (1,024 x 1,024 pixels, pixel size: 0.284 µm) with the confocal scanning system (Leica SP8, Leica Microsystems, Germany), and images were captured using Leica Application Suite X (LasX, Leica Microsystems). Typically, 18 tile images were captured per DVC, with each tile consisting of 6–8 images in a z-stack (z-step = 1 µm).

Images were further analysed in FIJI (ImageJ) by drawing polygon regions of interest (ROIs) delineating the whole DVC subdivisions (the AP, NTS, DMV, and 4thVep). Two to three slices were analysed from each animal and averaged within each mouse.

Numerical results were statistically analysed using GraphPad Prism 10, and rhythmicity in clock gene expression was assessed using CircWave v1.4 (Dr. Roelof Hut, http://www.euclock.org/).

Relation of CircWave-determined acrophases of gene expression to feeding time was measured as a food entrainment error (FEE). FEE was calculated as a difference between the experimentally observed and the expected acrophase, relative to early night TRF. For example, a FEE would equal 0, if the peak gene expression was advanced by exactly 6 h following a 6 h phase advance of feeding from early night TRF (ZT12-18) to late day TRF (ZT6-12).

### RT^2^ profiler PCR arrays

#### Tissue preparation.

Thirty mice were fed *ad libitum* and culled at six daily timepoints with 4-h intervals over 24 h (*n* = 5 per group) by an overdose of pentobarbital sodium (i.p.; *Experiment A*). An additional group of 10 animals was acutely food restricted at ZT10, with a subgroup of five culled after 6 h (at ZT16; fasted), and the remaining five regaining access to food for 2 h before being humanely killed at ZT18 (re-fed; *Experiment B*). All 40 brains were extracted from the skull in ice-cold Hanks’ balanced salt solution (HBSS, Sigma, UK). Hindbrains were cut using a vibroslicer (Campden Instruments, UK) into 250 µm-thick slices containing the DVC, from which the AP and the bilateral NTS were collected using a sample corer (Fine Science Tools, Germany; ID: 0.5 mm). Eighty samples of tissue were then flash-frozen over dry ice and stored at −80 °C.

#### RNA extraction and RT-qPCR.

Tissue was processed for RNA extraction using the ReliaPrep RNA Tissue Miniprep System (Promega, USA). Extracted RNA in RNase-free water was stored at −80 °C until reverse transcription with the High-Capacity RNA-to-cDNA Kit (Applied Biosystems, USA). Subsequently, cDNA was stored at −20 °C. The RT-qPCR reaction was performed using RT^2^ Profiler PCR Arrays (Qiagen, USA) in the Mouse Neurotransmitter Receptors configuration (GeneGlobe ID: PAMM-060Z) – a preloaded PCR plate with qPCR primers targeting 84 genes coding a range of neurotransmitter receptors. Thermal cycling and data collection were performed using QuantStudio3 (Invitrogen).

#### Analysis and statistics.

Results were then analysed according to the Livak method (2^−ΔΔCT^) with *Gapdh* as the reference gene and presented as relative target gene expression (RQ), where RQ = 1 indicates the mean expression of a gene of interest at ZT0 (*Experiment A*) or at *ad libitum* fed ZT16 (*Experiment B*). Genes were classified as not expressed if their mean Ct throughout six daily timepoints >35. RQ values were further analysed in GraphPad Prism 10 to evaluate significant variability of expression between groups, and sine-wave fitted with CircWave v1.4 to assess daily rhythmicity in gene expression.

Motif analysis was conducted using the Biopython package (v1.78) in Python (v3.9.9) [73]. Instances of the BMAL1 binding profile were assessed in the promoter and 5′ untranslated sequence of the genes, 1kb either side of the transcription start side, with a false negative rate of 0.1. The BMAL1 binding profile used was from the JASPAR database (9th release; matrix ID MA0603.1) and the sequences were collected from the eukaryotic promoter database [35–37].

### Feeding, drinking, and wheel running assessment

#### Behavioural protocol and TRF.

Thirty-two naïve *Bmal1lox* mice were single-housed in running wheel-equipped cages with a precision balance to monitor drinking and feeding activity (TSE Systems, Germany). Wheel running, drinking, and feeding activities were recorded using PhenoMaster software (TSE Systems) following a similar experimental set-up as reported before [74]. All mice were first monitored for at least 10 days in a 12:12-h LD cycle under *ad libitum* feeding conditions, and then their behavioural activities were recorded in constant darkness (DD) for a further 10 days. After this period, the 12:12-h LD was re-established for the rest of the experiment. Following another 10 day-long epoch, mice were divided into two TRF cohorts (*n* = 16 each) with different times of food presentation. The first group had unlimited access to food for the last 6 h of the light phase (TRF ZT6-12), whereas the second cohort was fed during the first 6 h of the night (TRF ZT12-18). Animals underwent TRF for 7 days before being culled at four daily timepoints (ZT0, 6, 12, and 18). Water was provided *ad libitum* throughout the entirety of all protocols. Brain tissue was further used for RNAscope (see: Fluorescent in situ hybridisation (RNAscope)).

#### Behavioural assessment following Bmal1 knock-out in DVC neurons.

Eighteen Bmal1lox mice were deeply anaesthetised with ketamine (70 mg/kg, i.p.) and medetomidine (0.5 mg/kg, i.p.) and placed in a stereotaxic frame with the head gently tilted down to facilitate access to the caudal brainstem. A small midline incision was made in the skin of the neck between the occipital crest and the first vertebra. The overlying muscles were retracted to expose the meningeal layer, which was then carefully incised with a 26G needle at the level of the obex. A borosilicate glass pipette filled with viral vector was mounted on the injector, angled 30° from vertical, and lowered bilaterally into the NTS (400 µm lateral to the obex, 250 µm deep) and into the AP (150 µm deep, midline, under direct visual guidance). Viral injections of pENN.AAV8.hSyn.HI.eGFP-Cre.WPRE.SV40 or pAAV1.hSyn.eGFP.WPRE.bGH (10^12^, Addgene, MA, USA) were delivered at 100 nl/min, with 200 nl infused into the AP and 250 nl into each NTS. Fast Green dye (1%, Sigma) was added to the vector solution to allow visual confirmation of accurate infusion into the AP. Following successful injections, the incision was sutured and antiseptic wound powder applied. Mice received atipamezole (1 mg/kg, i.p.) to reverse anaesthesia and meloxicam (5 mg/kg, s.c.) for analgesia.

After a 2–3 week recovery period, animals were placed in the TSE System (see above) and their behavioural activity was recorded under LD and DD conditions (food *ad libitum*), followed by LD with TRF during the final 6 h of the light phase (ZT6-12). At the end of the behavioural protocol, all animals were euthanised by overdose of pentobarbital sodium, and brains were flash-frozen and processed for histological verification of injection sites.

### Behavioural analysis and statistics

The circadian period and power (the amplitude of the rhythm) of the behavioural data were assessed with a chi-squared periodogram using Actimetrics Clocklab software (v6.1.10; Lafayette Instrument Company, Lafayette, IN, USA). Feeding bouts were also assessed in Clocklab software, using a 1 min threshold. Outliers in the feeding and drinking data were removed with an exclusion criterion of above 12SD from the mean average of an active period using R (R v4.4.1). Statistical analysis of data was performed in GraphPad Prism 10.

### NanoString nCounter

#### Tissue preparation and RNA extraction.

Another group of 32 naïve *Bmal1lox* mice was single housed with *ad libitum* access to food and water, under a 12:12 h LD cycle. Then, animals were divided into two cohorts (*n* = 16 each). The first was subjected to TRF from ZT0 to ZT6 (early day), whereas the second group was fed between ZT12 and ZT18 (early night). Following 6–7 days of TRF, animals were culled at four daily timepoints in 6-h intervals (ZT0, 6, 12, and 18). Tissue was processed in the same way as for RT-qPCR, and RNA extracted from 32 AP and 32 NTS tissue punches was stored at −80 °C.

#### Probe design, sample processing, and analysis.

For each reaction, we used a combination of 11 probes: seven targeting neurotransmitter receptors (*Htr2c, Grik5, Gabra4, Gabra1, Adra2a, Prokr2, Sstr1*), two - molecular clock components (*Arntl* and *Per2*) and two - housekeeping genes (*Actb* and *Gapdh*). Oligonucleotides were purchased separately from Integrated DNA Technologies (IDT, USA). Samples were processed by the Genomics Core Facility at the University of Bristol according to nCounter Elements TagSets protocol provided by NanoString. Data were analysed using nSolver 4.0 software (NanoString). Raw counts were first thresholded with a geometric mean of the negative control counts and then normalised to positive controls and housekeeping genes. Data were presented as relative target gene expression (RQ), where RQ = 1 indicates the mean expression of a gene of interest at ZT0. RQ values were further plotted and statistically analysed in GraphPad Prism 10, and sine-wave fitted with CircWave v1.4.

## Supporting information

S1 Fig Rhythmic expression of neurotransmitter receptor genes in the AP and NTS. Sine-wave fitted normalised counts of rhythmic neurotransmitter receptor transcripts in the AP and in the NTS. Data were collected using RT qPCR Profiler Arrays. RQ – gene expression relative to ZT0. *p < 0.05, **p < 0.01, Kruskal-Wallis test. The underlying data for this figure can be found in S1 Data.
(TIF)

S2 Fig Enrichment analysis of JASPAR transcription factor binding sites (TFBSs). (*left*) TFBS sequence motif for a putative E-box sequence is illustrated with the height of each base indicative of the probability of their presence at the designated position. (*right*) A list of genes showing results of the putative E-box sequence search.
(TIF)

S3 Fig Wheel running, food intake, and body weight change in mice subjected to late-day or early-night time-restricted feeding (TRF). **(A)** Average daily food intake and wheel running distance over 1 week under 12:12 light-dark (LD) conditions during *ad libitum* feeding, prior to TRF. **(B)** The same measures recorded during the TRF protocols. Red and black bars indicate the feeding windows for late-day and early-night fed mice, respectively. **(C)** Average food intake during the 6 h restricted feeding window. **(D)** Body weight change (%) relative to the start of the experiment, following the TRF schedule. (E) Average wheel running distance during the food-anticipatory activity (FAA) window (ZT0–6 for late-day fed mice and ZT6–12 for early-night fed mice) and during the nocturnal activity period (ZT12–24) for both groups. *p < 0.05, t *t*est, **p < 0.01, Sidak multiple comparisons test. The underlying data for this figure can be found in S1 Data.
(TIF)

S4 Fig Effects of a one-off food restriction on the transcription of neurotransmitter receptor genes in the DVC. The cartoon shows an experimental timeline – animals were food restricted between ZT10 and ZT16, and some of them were re-fed 2 h prior to the cull. Scatter plots show the result of RT qPCR Profiler Array comparison of relative gene expression (RQ, normalised to ZT16) between ZT16 with (black)and without (blue) access to food, and the refeeding condition (red). Kruskal-Walis test results are presented as *p* values in the graphs, whereas * show the result of Dunn’s multiple comparisons test. Only genes significantly changed by acute feeding conditions (as depicted by main ANOVA result) are presented. ~ next to the gene name depicts that this transcript was deemed to be rhythmic under *ad libitum* conditions, when evaluated over 24 h (see Fig 2). The underlying data for this figure can be found in S1 Data.
(TIF)

S5 Fig Summary of acrophases of *Bmal1*, *Per2*, and *Reverbα* expression in four parts of the DVC under different food presentation times. Raleigh plots display peak expression for core clock genes under *ad libitum* feeding (*top left*) in four time-restricted feeding (TRF) conditions. Arches within the Raleigh plots depict TRF time: orange – early day ZT0–6, magenta – late day ZT6–12, green – early night ZT12–18, and blue – late day ZT0–6. Note that genes which were not significantly rhythmic (CircWave *p* > 0.05 for a sine wave fit) were not plotted. Data were collected using area fraction measurements on images obtained with RNAscope in situ hybridisation. AP – area postrema, NTS – nucleus of the solitary tract, DMV – dorsal motor nucleus of the vagus, 4thVep – ependymal cell layer lining the 4th ventricle/central canal. The underlying data for this figure can be found in S1 Data.
(TIF)

S6 Fig Core clock gene expression in the DVC over 24 h under early day and late night time-restricted feeding (TRF). **(A, C)** Orange and blue bars depict the TRF epoch, whereas significant fit of a sine wave (CircWave *p* > 0.05) shows rhythmicity of a transcript across 24 h. Data were collected using area fraction measurements on images obtained with RNAscope in situ hybridisation. **(B, D)** Raleigh plots displaying acrophases for core clock genes in early day TRF, where the orange and blue arches depict the time of food presentation. Note that genes which were not significantly rhythmic were not plotted. **(E)** Food entrainment error relative to early night TRF (FEE = observed phase – projected phase) for *Bmal1* (blue), *Per2* (red), and *Reverbα* (purple) for late day (squares), early day (circles), and late night TRF (triangles). AP – area postrema, NTS – nucleus of the solitary tract, DMV – dorsal motor nucleus of the vagus, 4thVep – ependymal cell layer lining the 4th ventricle/central canal. The underlying data for this figure can be found in S1 Data.
(TIF)

S7 Fig Transfection range and body weights of mice following AAV-mediated neurone-specific *Bmal1* knockout in the DVC. **(A)** Nissl-stained section and corresponding atlas plate at the level of the mid-AP DVC (Allen Mouse Brain Reference Atlas, P56, coronal, image 128). **(B)** Representative brainstem section showing AAV-Syn-Cre:GFP transfection (GFP, green) within the DVC, with the AP, NTS, DMV, and fourth ventricle ependyma delineated. **(C)** Composite overlay of GFP transfection range across all animals. **(D)** Body weight across the experimental timeline. Left: absolute body weight at four timepoints — entry into the TSE system, after 2 weeks (onset of constant darkness, DD), after 25 days (end of DD and reintroduction of the light-dark cycle, LD), and after 35 days (completion of 1 week of time-restricted feeding, TRF). Right: body weight change (%) normalised to body weight on the day of surgery. The underlying data for this figure can be found in S1 Data.
(TIF)

S1 Data Summary of underlying data for all figures and supplementary figures. (XLSX)

## Abbreviations

AP: area postrema
AWERB: Animal Welfare and Ethics Review Body
CP: choroid plexus
DVC: dorsal vagal complex
FAA: food anticipatory activity
FEE: food entrainment error
FEO: food-entrainable oscillator
HFD: high-fat diet
IDT: Integrated DNA Technologies
LD: light-dark
PBS: phosphate buffered saline
ROIs: regions of interest
RT: room temperature
TFBSs: transcription factor binding sites
TRF: time-restricted feeding
TTFL: transcription-translation feedback loops
ZT: Zeitgeber time.
