# Supplementary material for: Meal timing entrains circadian rhythms in the dorsal vagal complex, a brainstem satiety centre
Source: PLoS Biol. 2026 Aug 4;24(8):e3003936. doi: 10.1371/journal.pbio.3003936 (PMC13460586; doi:10.1371/journal.pbio.3003936)
Supplement: Supplementary file 1 [file pbio.3003936.s011.docx]

Reviewer #1:

The dorsal vagal complex (DVC) is a key regulatory center for brain-body signaling particularly regarding food intake. The authors' previous work has indicated that the DVC is a cell-autonomous circadian oscillator, but it is unclear what rhythmic stimuli entrain the DVC clock. In the present work, Chrobok et al. find that the AP and NTS clock can entrain to time-restricted feeding protocols and have a unique set of clock-controlled genes indicating that specific DVC metabolic regulatory functions are under circadian control. They also found that the DVC on its own is not the food-entrainable oscillator but suggest that the DVC could act within a food-entrainable oscillatory network.
        This is an elegant and fascinating study from a group that continues to advance our understanding of timekeeping in the DVC. This is a valuable and important addition to the field, but there are a few points that I would like the authors to address before accepting for publication.

Thank you for your positive assessment of our study and for your enthusiasm. We have made every effort to respond to all of your comments, including addressing your major points by running additional experiments.

Major points

1.      I believe a late active period TRF experiment is lacking and necessary to fully characterize the sensitivity of the DVC clock to food timing. The results indicate that the DVC can entrain to a 6hr phase advance, but not to a 12hr phase delay. Rather than concluding that the DVC is unable to entrain to phase delays in general, DVC entrainment to a 6hr phase delay in food availability should be tested.

Following the reviewer’s suggestion, we ran an additional experiment in which mice were time-restricted fed for one week between ZT18 and ZT0 (late active period). The results of an RNAscope measurement of clock entrainment are now added to SFig. 5 and 6.

These new results strengthen our hypothesis that the DVC oscillators are more readily entrained to a phase advance than to a phase delay. To emphasise this, we have amended the main text in the Results:

*Similarly, another cohort of 14 mice underwent a TRF at late night (ZT18-0) for one week. RNAscope assessment of clock gene expression showed similar, partial misalignment of oscillators with Bmal1 and Per2 not significantly rhythmic in the AP, and Per2 not cycling in the NTS. In this cohort, only Bmal1 in the NTS and DMV consistently followed the mealtime (Supplementary Figure 6).*

*However, under an early day TRF (ZT0-6) and late night TRF (ZT18-0), only the acrophase of most clock gene expression in the NTS (with the exception of Reverbα in the late night TRF group) and Bmal1 in the DMV following the late night feeding showed a food entrainment error in this range, with all other structures exhibiting error in the -2h to -8h range (Supplementary Figure 6).*

And the Discussion section:

*A late-day TRF schedule (phase advance relative to the active phase) produced more reliable shifts in DVC rhythms compared to early-day or late-night TRF (phase delay). This aligns with behavioural evidence suggesting that circadian rhythms are more readily advanced by non-photic cues and delayed by photic stimuli [58].*

2.      The authors should include data from a peripheral tissue whose molecular clock is known to entrain to food timing under TRF conditions. This would ensure that the 1 week of TRF exposure was long enough to shift food-entrainable clocks. The liver would be a great choice, but other peripheral organs such as adipose or skeletal muscle would suffice.

To compare the rate of entrainment of the DVC clock with that of a peripheral oscillator, we performed an additional qPCR experiment on livers obtained from the new cohort of late-night-fed animals. We then compared this dataset with historical results from the same laboratory and under the same conditions, in which mice were culled at six daily timepoints following an ad libitum diet. Unfortunately, we had not collected peripheral tissues from the mice presented in the paper, and so were unable to perform a more thorough comparison.

However, we note that despite only partial entrainment of the DVC to the delayed feeding schedule, the liver clock was already fully entrained after one week of TRF.

Minor points

1.      In Figure 4B, put the early night TRF Raleigh plots to the right side and the late day TRF Raleigh plots to the left side of panel B. Magenta is on the left of green in panel A, so rearranging panel B to match makes it easier to interpret both blots side by side.

We agree with the reviewer. We have amended the figure, and it now reads better. Thank you.

Reviewer #2:

In this study, the authors demonstrate circadian rhythms of clock gene expression in the dorsal vagal complex (DVC) using Per2-luc bioluminescence imaging in slice preparations and in situ hybridization. They also show, by quantitative PCR, that the expression of many neurotransmitter receptor genes exhibits circadian rhythms. These expression rhythms are entrained by feeding. DVC-specific attenuation of molecular clock function prolonged the duration of individual feeding bouts, but did not affect the expression of food-entrainable circadian rhythms. The topic addressed in this study is important. However, I have concerns regarding the novelty and significance of the results obtained.

1. Overall, the study is largely descriptive in nature. Circadian rhythms of clock gene expression in the NTS and AP have already been reported, including by the authors' own group. While the circadian expression of neurotransmitter receptor genes may represent a novel finding, no information is provided regarding the physiological significance of these rhythms.

Thank you for this comment. We appreciate that exploring the physiological significance of these rhythms is beyond the scope of our study; here, we aimed to characterise a broad transcriptional landscape and to demonstrate that it is modulated in time by the food-entrained expression of clock genes. We hope that this general characterisation will inspire future, more targeted studies exploring the physiological roles of these distinct rhythms. Some speculation on the physiological role of these rhythms is provided in the Discussion section:

*Our findings have important implications for understanding the circadian plasticity of the DVC and its role in metabolic health. Feeding schedules that entrain DVC molecular clocks could optimise the timing of neurotransmitter receptor expression, potentially enhancing the efficacy of pharmacological interventions targeting DVC circuits. Pharmacological agents that target neurotransmitter receptors in the DVC including CNR1, HTR2c, and GLP1R are already in clinical use or under investigation for obesity and diabetes treatment [15,17,69]. Incorporating meal timing into chronopharmacological strategies could synergise with these treatments, utilising the DVC’s circadian rhythmicity to improve therapeutic outcomes.*

2. The DVC-specific Bmal1 knockdown represents the only functional manipulation in this study; however, it failed to produce any of the circadian clock-related phenotypes discussed earlier in this manuscript. The only reported effect is a statistically significant prolongation of feeding duration, limited to the light phase under LD conditions. However, the magnitude of this effect appears small. If this phenotype is physiologically meaningful, why is no abnormality observed under DD conditions? Moreover, there is no quantitative evaluation of the extent to which Bmal1 expression is actually reduced.

We agree with the reviewer’s comment: the effect size is small and is not reproducible under constant-darkness conditions, where feeding and other behavioural rhythms are generally less robust. We have toned down the discussion of these findings.

*The same genetic disruption of the DVC molecular clock moderately altered meal architecture, specifically lengthening meal bouts, but only during the light phase in LD conditions (…)*

*Due to a small effect size reported in our study, these experiments should be replicated in longer protocols and during the feeding challenge, e.g., with high-fat diet.*

Additionally, we have constructed a map showing the extent of the transfection in each animal (new SFig. 7A–C).

3. If feeding duration is prolonged by DVC-specific Bmal1 knockdown while feeding frequency remains unchanged, does this result in an increase in daily food intake? What about body weight?

Thank you for this question. Over the duration of our experiment, we did not observe significant changes in the amount of food eaten (see Figure 6C) or in the number of meals (see Figure 6E). This implies that other mechanisms must compensate for the prolonged meal length. Future experiments involving longer-term food-intake monitoring and a high-fat-diet challenge will certainly be needed.

The body-weight measurements have been added as SFig. 7D.

Reviewer #3:

The study by Piggins and colleagues extends their previous findings demonstrating circadian timekeeping across the dorsal vagal complex (DVC) and examines the rhythmicity of clock gene expression and consequent changes in mRNA expression levels of clock-regulated neurotransmitter receptor genes in DVC, under different feeding schemes. They utilize Per2:Luciferase reporter mice to show the presence of rhythmicity in DVC, focusing especially on AP and NTS, in ex vivo brain slices under ad libitum conditions, then show that two clock genes (Per2 and RevErbα, but not Bmal1) are rhythmic in the specified brain regions. They further analyze several neurotransmitter receptor transcripts and focus on the ones that are both rhythmically expressed and contain E-box elements for Bmal1 binding (although their findings show that Bmal1 is not rhythmic under ad libitum conditions). They show that both clock genes' and many of the neurotransmitter receptor genes' expression are
responsive to time restricted feeding (TRF), synchronizing to the feeding times. Finally, they show that ablating Bmal1 gene in AP and NTS does not affect either ad libitum food intake or food anticipatory activity (FAA) in response to TRF.
The findings of this study are important for understanding the circadian regulation of the DVC, which has been shown to be an important hub for regulation of satiety, metabolism and homeostatic functions. The finding that both the clock genes and the neurotransmitter receptor genes are synchronized to food timing as a cue rather than the light cues emphasizes its importance in food metabolism. In general, I find the study interesting and valuable, with only minor points to note.

Thank you for your positive response to our manuscript.

1. In the study, it's shown that Bmal1 gene is not rhythmic in the DVC under ad libitum conditions; however, when narrowing down the list of neurotransmitter receptor genes to study, authors select the ones that bear the Bmal1 E-box elements in their promoters to further analyze. Bmal1 is one of the most crucial transcription factors of the master clock, yet it still needs a justification to select the binding element for the only non-rhythmic clock protein tested, instead of the other two rhythmic proteins.

This is indeed an intriguing result and a limitation of our study. We hypothesise that Bmal1 rhythms in this cohort of ad libitum-fed animals were desynchronised between single-cell oscillators and were therefore not captured at the whole-tissue level, owing to a smaller effect size than for Per2 or Reverba. In a previous study performed at a different university (a different animal facility) and using a different method (RT-qPCR on laser-captured DVC slices), we were able to characterise significant Bmal1 rhythms in both the AP and NTS (see: https://doi.org/10.1038/s42003-020-0960-y).

2.      It is interesting to see that Bmal1 gene ablation in the NTS and AP does not have any impact on feeding behavior and FAA. It would be even more interesting to see how the rhythmicity of the neurotransmitter receptor genes (or other clock genes) are affected in these mice.

We agree with the reviewer that this is an interesting experiment. However, it would require a large additional number of mice undergoing brainstem surgery, which is unfortunately not feasible for us to perform at this time.

3.      Authors suggest that "rostral NTS regions lacked detectable bioluminescence" in Fig1A; however, in the representative images they have provided, bioluminescence is seen in center and rostral NTS, while less luminescence is seen in the most caudal regions. This need clarification.

By rostral NTS, we mean the portion of the DVC that lies rostral to the AP (the NTS extends considerably in the latero-rostral direction). The lack of bioluminescence in the rostral NTS can also be seen in the coronal section below: the NTS extends laterally to a ‘V’-shaped fourth ventricle (4V), which is occupied by the choroid plexus (ChP).

A clarification has been added to the Results section:

*(…) but in contrast, the NTS regions rostral to the AP lacked detectable bioluminescence.*

4.      Also, in Fig 1E, legend should be corrected to change second (D) to (E).

Thank you; this has been corrected.

5.      Although several neurotransmitter receptor genes have been analyzed and found to have rhythmicity that synchronized to TRF, discussion of the implications of these findings is limited.

Thank you for this comment. We appreciate that exploring the physiological significance of these rhythms is beyond the scope of our study; here, we aimed to characterise a broad transcriptional landscape and to demonstrate that it is modulated in time by the food-entrained expression of clock genes. We hope that this general characterisation will inspire future, more targeted studies exploring the physiological roles of these distinct rhythms. Some speculation on the physiological role of these rhythms is provided in the Discussion section (in green, as Reviewer 2 also requested):

*Our findings have important implications for understanding the circadian plasticity of the DVC and its role in metabolic health. Feeding schedules that entrain DVC molecular clocks could optimise the timing of neurotransmitter receptor expression, potentially enhancing the efficacy of pharmacological interventions targeting DVC circuits. Pharmacological agents that target neurotransmitter receptors in the DVC including CNR1, HTR2c, and GLP1R are already in clinical use or under investigation for obesity and diabetes treatment [15,17,69]. Incorporating meal timing into chronopharmacological strategies could synergise with these treatments, utilising the DVC’s circadian rhythmicity to improve therapeutic outcomes.*

6.      The study focuses on the regulation of circadian elements in response to food timing, and mentions the importance of synchronization of neural feeding circuits to availability of food, but fails to mention up to date literature on the topic, which delved into circuit mechanisms and showed the synchronization of neuron activity in hypothalamus to the feeding times (e.g. PMID: 39719709, PMID: 37957320, PMID: 37624889).

Thank you for pointing out these studies, which we had unintentionally omitted. They have now been added to the amended version of the manuscript.

Reviewer #4:

This is a well-designed and executed set of descriptive and experimental studies that confirm circadian rhythmicity intrinsic to multiple components of the DVC, identify a host of neurotransmitter genes under clock control, and establish phase control of these rhythms by daily feeding schedules. The manuscript is clearly written and the figures communicate the results effectively.  I have a few suggestions and comments for the authors to consider.

Thank you for your positive response to our study. We have amended the manuscript in line with your comments below.

1.      In section 3 of the Results, the authors write  "To assess how induction of the FEO influences DVC timekeeping, we monitored the feeding, drinking, and wheel-running behaviours of mice subjected to time-restricted feeding (TRF)."  In this context, the 'FEO' represent a putative food-entrainable oscillator(s) that drives food anticipatory activity rhythms induced by a daily feeding schedule. However, as established by this study, the DVC also contains FEOs (as do other brain regions and many other organs), and these may be entrained by feeding related cues, rather than by FEOs that control behavioural rhythms. There is presently no compelling evidence for a 'master' FEO comparable to the SCN light-entrainable clock that serves as a 'conductor' necessary for synchronizing FEOs in other locations.

We agree that this sentence is awkward; it has been amended to read: *To assess how feeding schedules influence DVC timekeeping (…).*

We would also like to stress that we share the reviewer’s view that there is no compelling evidence for a central, single-locus localisation of the FEO, and we hope that our study strengthens the evidence for the FEO’s distributed nature. Our DVC Bmal1KO experiment, in particular, provides further evidence that depleting rhythmic clock-gene expression in one of the food-entrained oscillators (in our case, the brainstem) does not abolish behavioural FAA.

2.      To determine whether DVC clocks and genes are entrained by a daily feeding schedule, mice were provided food for 6h/day for 7 days, with mealtimes beginning 6h after lights on (ZT6) or at lights-off (ZT12). Mice in the ZT6 group showed a robust bout of activity prior to mealtime, whereas mice in the ZT12 group did not. This difference was not explored, and I'm wondering if might be related to a group difference in the amount of food intake during the 6h of food available, and effects of that on body weight, which has a major effect on the amount of food anticipatory activity. Mice fed in the middle of the light period would most likely eat less and lose more weight than mice fed at the beginning of the night.  A third group of mice received a 6h daily meal beginning at lights-on (ZT0), and these mice showed differences in a 'food entrainment error' metric related to shifting of clock gene cycles. Differences in food intake and body weight (metabolic impact) associated with the 3 different mealtimes could potentially contribute to differences in this metric. As a general rule, such differences between mealtimes dissipate or disappear after several weeks of restricted feeding.

Owing to the limited availability of the behavioural phenotyping equipment, we measured food and water intake only in mice time-restricted fed between ZT6–ZT12 and ZT12–ZT18.

Indeed, the FAA of mice fed during the night was significantly lower than that of mice fed during the day (only 4 of 16 mice displayed notable FAA; we therefore chose to show, as a panel in the main figure, a representative animal that does not exhibit a significant rise in running-wheel activity). Interestingly, there were no differences in nocturnal wheel running during the scheduled feeding regime, highlighting the independence of these two behaviours: SCN-driven nocturnal wheel running and FEO-driven FAA wheel running.

However, our new comparison of food intake between late-day- and early-night-fed animals confirms the reviewer’s prediction: food presented at the ‘physiological’ time (at night) leads to significantly higher consumption. Interestingly, we observed no significant change in body weight, and no significant difference in weight change following TRF between the late-day- and early-night-fed cohorts.

Additional analyses of the food intake, FAA, and body weight of these animals have been added to the manuscript and SFig. 4, and are expanded upon in the Results section:

*FAA was significantly reduced in night-fed compared to day-fed animals (p=0.0134, Sidak multiple comparisons test, Supplementary Figure 4). Indeed, only 4 of 16 night-fed mice displayed notable FAA, consistent with feeding at the physiological time of day attenuating the anticipatory drive [38]. Nocturnal wheel running was unaffected by feeding schedule (p=0.1847, Sidak multiple comparisons test, Supplementary Figure 4), highlighting the behavioural independence of SCN-driven nocturnal activity and FEO-driven FAA. As predicted, food intake was significantly higher in night-fed animals (p=0.0074, t-test, Supplementary Figure 4). Despite this, no significant differences in body weight change following TRF were observed between the late-day and early-night fed cohorts (p=0.4778, t-test, Supplementary Figure 4).*

3.      At the end of section 3 of the results, the authors conclude that "the findings demonstrate that food-entrained rhythms in molecular clock genes drive corresponding shifts in the daily oscillations of secondary genes, ensuring that neurotransmitter receptor expression in the DVC is synchronised with feeding behaviour".  However, it seems possible that daily rhythms in some of these secondary genes could be driven by feeding-related inputs independently of circadian clock genes, and that to establish a causal relationship, the analyses would need to include clock gene knockout mice.

We agree that we cannot imply causation where our results show only a correlation. The term ‘drive’ has now been changed to ‘are accompanied by’.
