# Supplementary material for: Meal timing entrains circadian rhythms in the dorsal vagal complex, a brainstem satiety centre
Source: PLoS Biol. 2026 Aug 4;24(8):e3003936. doi: 10.1371/journal.pbio.3003936 (PMC13460586; doi:10.1371/journal.pbio.3003936)
Supplement: Peer review history

# Editor report: Decision Letter 0

**DOI:** 10.1371/journal.pbio.3003936.r001

**Authors:** Lucas Smith

9 Jan 2026

Dear Lukasz,

Thank you for submitting your manuscript entitled "Food-entrainment of circadian timekeeping in the dorsal vagal complex" for consideration as a Research Article by PLOS Biology. Please accept my apologies for the delay in sending you an initial decision for your paper - the PLOS Biology editorial office was closed over the Holidays, and I am just getting caught up now.

I have now had a chance to discuss your manuscript, which has been expanded in response to some editorial feedback that we gave on a previous version of this study, with an Academic Editor with relevant expertise and I am writing to let you know that we would like to send your submission out for external peer review.

However, before we can send your manuscript to reviewers, we need you to complete your submission by providing the metadata that is required for full assessment. To this end, please login to Editorial Manager where you will find the paper in the 'Submissions Needing Revisions' folder on your homepage. Please click 'Revise Submission' from the Action Links and complete all additional questions in the submission questionnaire.

Once your full submission is complete, your paper will undergo a series of checks in preparation for peer review. After your manuscript has passed the checks it will be sent out for review. To provide the metadata for your submission, please Login to Editorial Manager (https://www.editorialmanager.com/pbiology) within two working days, i.e. by Jan 13 2026 11:59PM.

If your manuscript has been previously peer-reviewed at another journal, PLOS Biology is willing to work with those reviews in order to avoid re-starting the process. Submission of the previous reviews is entirely optional and our ability to use them effectively will depend on the willingness of the previous journal to confirm the content of the reports and share the reviewer identities. Please note that we reserve the right to invite additional reviewers if we consider that additional/independent reviewers are needed, although we aim to avoid this as far as possible. In our experience, working with previous reviews does save time.

If you would like us to consider previous reviewer reports, please edit your cover letter to let us know and include the name of the journal where the work was previously considered and the manuscript ID it was given. In addition, please upload a response to the reviews as a 'Prior Peer Review' file type, which should include the reports in full and a point-by-point reply detailing how you have or plan to address the reviewers' concerns.

During the process of completing your manuscript submission, you will be invited to opt-in to posting your pre-review manuscript as a bioRxiv preprint. Visit http://journals.plos.org/plosbiology/s/preprints for full details. If you consent to posting your current manuscript as a preprint, please upload a single Preprint PDF.

Feel free to email us at plosbiology@plos.org if you have any queries relating to your submission.

Kind regards,

Luke

Lucas Smith, Ph.D.

Senior Editor

PLOS Biology

lsmith@plos.org

# Peer review: Decision Letter 1

**DOI:** 10.1371/journal.pbio.3003936.r002

**Authors:** Lucas Smith

19 Feb 2026

Dear Lukasz,

Thank you for your patience while your manuscript "Food-entrainment of circadian timekeeping in the dorsal vagal complex" was peer-reviewed at PLOS Biology. Please accept my apologies for the protracted review process, which was caused, in part, by the closer of the PLOS Biology offices over the Christmas Holiday. Your study has now been evaluated by the PLOS Biology editors, an Academic Editor with relevant expertise, and by several independent reviewers.

In light of the reviews, which you will find at the end of this email, we would like to invite you to revise the work to thoroughly address the reviewers' reports.

As you will see below, the reviewers generally find the work interesting and well done although they have also raised a number of important suggestions to strengthen the study further. We think that, in general, the reviewer concerns will need to be thoroughly addressed before we can consider your study for publication. This will require you to add analyses to further support and flesh out your conclusions - although in places (ex Reviewer 4's point 3) we think the reviewer comments can be addressed by toning down conclusions and/or discussing alternative interpretations of the data.

Considering the positive feedback from reviewers, I would add that, editorially, we do not share reviewer 2's view that the paper does not offer the level of novelty and significance needed for publication at PLOS Biology. However, we do agree with this reviewer that it would be important to further test the physiological significance of the rhythms reported here - by, for example, reporting what happens to body weight when frequency of food intake is unchanged but duration is increased. If possible, we encourage you to add that analysis.

If it would be helpful, we would be happy to discuss the reviews further and to provide feedback on a revision plan. If that is of interest to you, please reach out over email.

Given the extent of revision needed, we cannot make a decision about publication until we have seen the revised manuscript and your response to the reviewers' comments. Your revised manuscript is likely to be sent for further evaluation by all or a subset of the reviewers.

In addition to these revisions, you will need to complete some formatting changes, which you will receive in a follow up email. A member of our team will be in touch with a set of requests shortly.

We expect to receive your revised manuscript within 3 months. Please email us (plosbiology@plos.org) if you have any questions or concerns, or would like to request an extension.

At this stage, your manuscript remains formally under active consideration at our journal; please notify us by email if you do not intend to submit a revision so that we may withdraw it.

**IMPORTANT - SUBMITTING YOUR REVISION**

Your revisions should address the specific points made by each reviewer. Please submit the following files along with your revised manuscript:

1. A 'Response to Reviewers' file - this should detail your responses to the editorial requests, present a point-by-point response to all of the reviewers' comments, and indicate the changes made to the manuscript.

*NOTE: In your point-by-point response to the reviewers, please provide the full context of each review. Do not selectively quote paragraphs or sentences to reply to. The entire set of reviewer comments should be present in full and each specific point should be responded to individually, point by point.

You should also cite any additional relevant literature that has been published since the original submission and mention any additional citations in your response.

2. In addition to a clean copy of the manuscript, please also upload a 'track-changes' version of your manuscript that specifies the edits made. This should be uploaded as a "Revised Article with Changes Highlighted" file type.

*Re-submission Checklist*

When you are ready to resubmit your revised manuscript, please refer to this re-submission checklist: https://plos.io/Biology_Checklist

To submit a revised version of your manuscript, please go to https://www.editorialmanager.com/pbiology/ and log in as an Author. Click the link labelled 'Submissions Needing Revision' where you will find your submission record.

Please make sure to read the following important policies and guidelines while preparing your revision:

*Published Peer Review*

Please note while forming your response, if your article is accepted, you may have the opportunity to make the peer review history publicly available. The record will include editor decision letters (with reviews) and your responses to reviewer comments. If eligible, we will contact you to opt in or out. Please see here for more details:

https://blogs.plos.org/plos/2019/05/plos-journals-now-open-for-published-peer-review/

*PLOS Data Policy*

Please note that as a condition of publication PLOS' data policy (http://journals.plos.org/plosbiology/s/data-availability) requires that you make available all data used to draw the conclusions arrived at in your manuscript. If you have not already done so, you must include any data used in your manuscript either in appropriate repositories, within the body of the manuscript, or as supporting information (N.B. this includes any numerical values that were used to generate graphs, histograms etc.). For an example see here: http://www.plosbiology.org/article/info%3Adoi%2F10.1371%2Fjournal.pbio.1001908#s5

*Blot and Gel Data Policy*

We require the original, uncropped and minimally adjusted images supporting all blot and gel results reported in an article's figures or Supporting Information files. We will require these files before a manuscript can be accepted so please prepare them now, if you have not already uploaded them. Please carefully read our guidelines for how to prepare and upload this data: https://journals.plos.org/plosbiology/s/figures#loc-blot-and-gel-reporting-requirements

*Protocols deposition*

To enhance the reproducibility of your results, we recommend that if applicable you deposit your laboratory protocols in protocols.io, where a protocol can be assigned its own identifier (DOI) such that it can be cited independently in the future. Additionally, PLOS ONE offers an option for publishing peer-reviewed Lab Protocol articles, which describe protocols hosted on protocols.io. Read more information on sharing protocols at https://plos.org/protocols?utm_medium=editorial-email&utm_source=authorletters&utm_campaign=protocols

Thank you again for your submission to our journal. We hope that our editorial process has been constructive thus far, and we welcome your feedback at any time. Please don't hesitate to contact us if you have any questions or comments.

Sincerely,

Luke

Lucas Smith, Ph.D.

Senior Editor

PLOS Biology

lsmith@plos.org

------------------------------------

REVIEWS:

Reviewer #1: COMMENTS TO AUTHORS

The dorsal vagal complex (DVC) is a key regulatory center for brain-body signaling particularly regarding food intake. The authors' previous work has indicated that the DVC is a cell-autonomous circadian oscillator, but it is unclear what rhythmic stimuli entrain the DVC clock. In the present work, Chrobok et al. find that the AP and NTS clock can entrain to time-restricted feeding protocols and have a unique set of clock-controlled genes indicating that specific DVC metabolic regulatory functions are under circadian control. They also found that the DVC on its own is not the food-entrainable oscillator but suggest that the DVC could act within a food-entrainable oscillatory network.

This is an elegant and fascinating study from a group that continues to advance our understanding of timekeeping in the DVC. This is a valuable and important addition to the field, but there are a few points that I would like the authors to address before accepting for publication.

Major points

1.I believe a late active period TRF experiment is lacking and necessary to fully characterize the sensitivity of the DVC clock to food timing. The results indicate that the DVC can entrain to a 6hr phase advance, but not to a 12hr phase delay. Rather than concluding that the DVC is unable to entrain to phase delays in general, DVC entrainment to a 6hr phase delay in food availability should be tested.

2.The authors should include data from a peripheral tissue whose molecular clock is known to entrain to food timing under TRF conditions. This would ensure that the 1 week of TRF exposure was long enough to shift food-entrainable clocks. The liver would be a great choice, but other peripheral organs such as adipose or skeletal muscle would suffice.

Minor points

1.In Figure 4B, put the early night TRF Raleigh plots to the right side and the late day TRF Raleigh plots to the left side of panel B. Magenta is on the left of green in panel A, so rearranging panel B to match makes it easier to interpret both blots side by side.

Reviewer #2: In this study, the authors demonstrate circadian rhythms of clock gene expression in the dorsal vagal complex (DVC) using Per2-luc bioluminescence imaging in slice preparations and in situ hybridization. They also show, by quantitative PCR, that the expression of many neurotransmitter receptor genes exhibits circadian rhythms. These expression rhythms are entrained by feeding. DVC-specific attenuation of molecular clock function prolonged the duration of individual feeding bouts, but did not affect the expression of food-entrainable circadian rhythms. The topic addressed in this study is important. However, I have concerns regarding the novelty and significance of the results obtained.

Overall, the study is largely descriptive in nature. Circadian rhythms of clock gene expression in the NTS and AP have already been reported, including by the authors' own group. While the circadian expression of neurotransmitter receptor genes may represent a novel finding, no information is provided regarding the physiological significance of these rhythms.

The DVC-specific Bmal1 knockdown represents the only functional manipulation in this study; however, it failed to produce any of the circadian clock-related phenotypes discussed earlier in this manuscript. The only reported effect is a statistically significant prolongation of feeding duration, limited to the light phase under LD conditions. However, the magnitude of this effect appears small. If this phenotype is physiologically meaningful, why is no abnormality observed under DD conditions? Moreover, there is no quantitative evaluation of the extent to which Bmal1 expression is actually reduced.

If feeding duration is prolonged by DVC-specific Bmal1 knockdown while feeding frequency remains unchanged, does this result in an increase in daily food intake? What about body weight?

Reviewer #3: The study by Piggins and colleagues extends their previous findings demonstrating circadian timekeeping across the dorsal vagal complex (DVC) and examines the rhythmicity of clock gene expression and consequent changes in mRNA expression levels of clock-regulated neurotransmitter receptor genes in DVC, under different feeding schemes. They utilize Per2:Luciferase reporter mice to show the presence of rhythmicity in DVC, focusing especially on AP and NTS, in ex vivo brain slices under ad libitum conditions, then show that two clock genes (Per2 and RevErbα, but not Bmal1) are rhythmic in the specified brain regions. They further analyze several neurotransmitter receptor transcripts and focus on the ones that are both rhythmically expressed and contain E-box elements for Bmal1 binding (although their findings show that Bmal1 is not rhythmic under ad libitum conditions). They show that both clock genes' and many of the neurotransmitter receptor genes' expression are responsive to time restricted feeding (TRF), synchronizing to the feeding times. Finally, they show that ablating Bmal1 gene in AP and NTS does not affect either ad libitum food intake or food anticipatory activity (FAA) in response to TRF.

The findings of this study are important for understanding the circadian regulation of the DVC, which has been shown to be an important hub for regulation of satiety, metabolism and homeostatic functions. The finding that both the clock genes and the neurotransmitter receptor genes are synchronized to food timing as a cue rather than the light cues emphasizes its importance in food metabolism. In general, I find the study interesting and valuable, with only minor points to note.

1.In the study, it's shown that Bmal1 gene is not rhythmic in the DVC under ad libitum conditions; however, when narrowing down the list of neurotransmitter receptor genes to study, authors select the ones that bear the Bmal1 E-box elements in their promoters to further analyze. Bmal1 is one of the most crucial transcription factors of the master clock, yet it still needs a justification to select the binding element for the only non-rhythmic clock protein tested, instead of the other two rhythmic proteins.

2.It is interesting to see that Bmal1 gene ablation in the NTS and AP does not have any impact on feeding behavior and FAA. It would be even more interesting to see how the rhythmicity of the neurotransmitter receptor genes (or other clock genes) are affected in these mice.

3.Authors suggest that "rostral NTS regions lacked detectable bioluminescence" in Fig1A; however, in the representative images they have provided, bioluminescence is seen in center and rostral NTS, while less luminescence is seen in the most caudal regions. This need clarification.

4.Also, in Fig 1E, legend should be corrected to change second (D) to (E).

5.Although several neurotransmitter receptor genes have been analyzed and found to have rhythmicity that synchronized to TRF, discussion of the implications of these findings is limited.

6.The study focuses on the regulation of circadian elements in response to food timing, and mentions the importance of synchronization of neural feeding circuits to availability of food, but fails to mention up to date literature on the topic, which delved into circuit mechanisms and showed the synchronization of neuron activity in hypothalamus to the feeding times (e.g. PMID: 39719709, PMID: 37957320, PMID: 37624889).

Reviewer #4: This is a well-designed and executed set of descriptive and experimental studies that confirm circadian rhythmicity intrinsic to multiple components of the DVC, identify a host of neurotransmitter genes under clock control, and establish phase control of these rhythms by daily feeding schedules. The manuscript is clearly written and the figures communicate the results effectively. I have a few suggestions and comments for the authors to consider.

1.In section 3 of the Results, the authors write "To assess how induction of the FEO influences DVC timekeeping, we monitored the feeding, drinking, and wheel-running behaviours of mice subjected to time-restricted feeding (TRF)." In this context, the 'FEO' represent a putative food-entrainable oscillator(s) that drives food anticipatory activity rhythms induced by a daily feeding schedule. However, as established by this study, the DVC also contains FEOs (as do other brain regions and many other organs), and these may be entrained by feeding related cues, rather than by FEOs that control behavioural rhythms. There is presently no compelling evidence for a 'master' FEO comparable to the SCN light-entrainable clock that serves as a 'conductor' necessary for synchronizing FEOs in other locations.

2.To determine whether DVC clocks and genes are entrained by a daily feeding schedule, mice were provided food for 6h/day for 7 days, with mealtimes beginning 6h after lights on (ZT6) or at lights-off (ZT12). Mice in the ZT6 group showed a robust bout of activity prior to mealtime, whereas mice in the ZT12 group did not. This difference was not explored, and I'm wondering if might be related to a group difference in the amount of food intake during the 6h of food available, and effects of that on body weight, which has a major effect on the amount of food anticipatory activity. Mice fed in the middle of the light period would most likely eat less and lose more weight than mice fed at the beginning of the night. A third group of mice received a 6h daily meal beginning at lights-on (ZT0), and these mice showed differences in a 'food entrainment error' metric related to shifting of clock gene cycles. Differences in food intake and body weight (metabolic impact) associated with the 3 different mealtimes could potentially contribute to differences in this metric. As a general rule, such differences between mealtimes dissipate or disappear after several weeks of restricted feeding.

3.At the end of section 3 of the results, the authors conclude that "the findings demonstrate that food-entrained rhythms in molecular clock genes drive corresponding shifts in the daily oscillations of secondary genes, ensuring that neurotransmitter receptor expression in the DVC is synchronised with feeding behaviour". However, it seems possible that daily rhythms in some of these secondary genes could be driven by feeding-related inputs independently of circadian clock genes, and that to establish a causal relationship, the analyses would need to include clock gene knockout mice.

# Author comment: Author response to Decision Letter 2

**DOI:** 10.1371/journal.pbio.3003936.r003

15 Jul 2026

Attachment Submitted filename: final_response_rev_v2.docx

# Editor report: Decision Letter 2

**DOI:** 10.1371/journal.pbio.3003936.r004

**Authors:** Lucas Smith

21 Jul 2026

Dear Lukasz,

Thank you for the submission of your revised Research Article "Food-entrainment of circadian timekeeping in the dorsal vagal complex" for publication in PLOS Biology. Your revision has been assessed by the PLOS Biology editorial staff, and by the Academic Editor, Samer Hattar, who is fully satisfied by the revision and thinks you have adequately addressed the previous reviewer concerns. Given this assessment, I am pleased to say that we can in principle accept your manuscript for publication, provided you address any remaining formatting and reporting issues. These will be detailed in an email you should receive within 2-3 business days from our colleagues in the journal operations team; no action is required from you until then. Please note that we will not be able to formally accept your manuscript and schedule it for publication until you have completed any requested changes.

**IMPORTANT - in addition to addressing and production related requests, to come, we also ask that you address the following editorial requests:

1) TITLE: After some discussion with the team, we would like to propose a tweak to the title to make it more active and accessible to a broad audience. If you agree we suggest you change the title to:

Meal timing entrains circadian rhythms in the dorsal vagal complex brainstem satiety center.

^We are happy to discuss further if helpful

2 - DATA AVAILABILITY: We generally do not allow 'data available upon request' in our data availability statements - but I see that you have already provided the necessary underlying data for your study as an excel file, and this does meet our reporting requirements. I would ask that you maybe adjust the data availability statement to read:

All relevant data are within the paper and its Supporting Information files. Additional raw data are available upon reasonable request (data-bris@bristol.ac.uk).

3 - DATA AVAILABILITY: Please rename your supplemental data file (currently open_data_figures) to 'S1_data'. Please also add a sentence to each figure legend directing readers to the underlying data. For example you can add the sentence 'The underlying data for this figure can be found in S1_data'.

4 - CODE: Did you generate any code for this study? If so, please make it available without restrictions. Please ensure that the code is sufficiently well documented and reusable, and that your Data Statement in the Editorial Manager submission system accurately describes where your code can be found. More information on our Code Policy, what and how to share can be found here: https://journals.plos.org/plosbiology/s/code-availability

Please note that we cannot accept sole deposition of code in GitHub, as this could be changed after publication. However, you can archive this version of your publicly available GitHub code to Zenodo. Once you do this, it will generate a DOI number, which you will need to provide in the Data Accessibility Statement (you are welcome to also provide the GitHub access information). See the process for doing this here: https://docs.github.com/en/repositories/archiving-a-github-repository/referencing-and-citing-content

Please take a minute to log into Editorial Manager at http://www.editorialmanager.com/pbiology/, click the "Update My Information" link at the top of the page, and update your user information to ensure an efficient production process.

PRESS

We frequently collaborate with press offices. If your institution or institutions have a press office, please notify them about your upcoming paper at this point, to enable them to help maximise its impact. If the press office is planning to promote your findings, we would be grateful if they could coordinate with biologypress@plos.org. If you have previously opted in to the early version process, we ask that you notify us immediately of any press plans so that we may opt out on your behalf.

We also ask that you take this opportunity to read our Embargo Policy regarding the discussion, promotion and media coverage of work that is yet to be published by PLOS. As your manuscript is not yet published, it is bound by the conditions of our Embargo Policy. Please be aware that this policy is in place both to ensure that any press coverage of your article is fully substantiated and to provide a direct link between such coverage and the published work. For full details of our Embargo Policy, please visit http://www.plos.org/about/media-inquiries/embargo-policy/.

Thank you again for choosing PLOS Biology for publication and supporting Open Access publishing. We look forward to publishing your study.

Sincerely,

Luke

Lucas Smith, Ph.D.

Senior Editor

PLOS Biology

lsmith@plos.org
